# scMelody: An Enhanced Consensus-Based Clustering Model for Single-Cell Methylation Data by Reconstructing Cell-to-Cell Similarity

**DOI:** 10.3389/fbioe.2022.842019

**Published:** 2022-02-23

**Authors:** Qi Tian, Jianxiao Zou, Jianxiong Tang, Liang Liang, Xiaohong Cao, Shicai Fan

**Affiliations:** ^1^ School of Automation Engineering, University of Electronic Science and Technology of China, Chengdu, China; ^2^ Intelligent Terminal Key Laboratory of Sichuan Province, University of Electronic Science and Technology of China, Chengdu, China; ^3^ Shenzhen Institute for Advanced Study, University of Electronic Science and Technology of China, Shenzhen, China; ^4^ Cancer Center, Sichuan Provincial People’s Hospital, University of Electronic Science and Technology of China, Chengdu, China; ^5^ Department of Geriatric Endocrinology, Sichuan Provincial People’s Hospital, University of Electronic Science and Technology of China, Chengdu, China

**Keywords:** single-cell, DNA methylation, epigenetic heterogeneity, consensus-based clustering, cell-to-cell similarity

## Abstract

Single-cell DNA methylation sequencing technology has brought new perspectives to investigate epigenetic heterogeneity, supporting a need for computational methods to cluster cells based on single-cell methylation profiles. Although several methods have been developed, most of them cluster cells based on single (dis)similarity measures, failing to capture complete cell heterogeneity and resulting in locally optimal solutions. Here, we present scMelody, which utilizes an enhanced consensus-based clustering model to reconstruct cell-to-cell methylation similarity patterns and identifies cell subpopulations with the leveraged information from multiple basic similarity measures. Besides, benefitted from the reconstructed cell-to-cell similarity measure, scMelody could conveniently leverage the clustering validation criteria to determine the optimal number of clusters. Assessments on distinct real datasets showed that scMelody accurately recapitulated methylation subpopulations and outperformed existing methods in terms of both cluster partitions and the number of clusters. Moreover, when benchmarking the clustering stability of scMelody on a variety of synthetic datasets, it achieved significant clustering performance gains over existing methods and robustly maintained its clustering accuracy over a wide range of number of cells, number of clusters and CpG dropout proportions. Finally, the real case studies demonstrated the capability of scMelody to assess known cell types and uncover novel cell clusters.

## 1 Introduction

As a heritable covalent chemical modification, DNA methylation is closely correlated with cell growth, differentiation, and transformation, which plays decisive roles in diseases and tumorigenesis (Aran and Hellman, 2013; Oakes et al., 2016; Koch et al., 2018). Technological advances have enabled DNA methylation assay at single-nucleotide resolution through high-throughput sequencing (Cokus et al., 2008; Sandoval et al., 2011; Krueger et al., 2012), thus paving the way for quantifying the methylation landscapes across different tissues and individuals. However, bulk protocols typically require thousands to millions of cells per experiment, making it difficult to study rare cell populations or explore the intercellular epigenetic heterogeneity (Schwartzman and Tanay, 2015). With increasing evidence of epigenetic heterogeneity in phenotypically similar cells (Angermueller et al., 2016; Hui et al., 2018), the single-cell methylation sequencing (scME-seq) protocols have demonstrated their capability for the deconvolution of mixed cell populations, such as scBS (Smallwood et al., 2014), scRRBS (Guo et al., 2013), and scWGBS (Farlik et al., 2015). Besides, the parallel single-cell sequencing protocols, like scM&T-seq (Angermueller et al., 2016), scTrio-seq (Hou et al., 2016), and scNOMe-seq (Pott, 2017), have brought new sights into understanding the regulatory mechanisms of epigenetic modifications on transcriptional variation. Although single-cell RNA sequencing (scRNA-Seq) has been widely used for investigating cell heterogeneity, it mainly informs about highly expressed transcripts while scME-seq enables detecting the methylation status of CpGs across gene and non-gene regions (Luo et al., 2017). Moreover, DNA methylation landscapes are not affected by the environment and can be more stable over the lifespan (Lister et al., 2013; Mo et al., 2015). Therefore, how to uncover cellular heterogeneity based on single-cell methylation data is gaining more attention.

To our knowledge, most existing methods incorporated different (dis)similarity relationships between cells into the distance-based clustering algorithms, such as hierarchical clustering (HC), to generate cell partitions. For instance, Farlik et al. clustered cells based on the average methylation over putative regulatory regions using HC with Euclidean distance and complete linkage (Farlik et al., 2016). Besides, a sliding window approach (Smallwood et al., 2014) was proposed to estimate CpG methylation rates and then cells were clustered based on the estimated methylation levels of most variable CpGs (Smallwood et al., 2014) or gene bodies (Angermueller et al., 2016) using Euclidean distance and HC. In addition to the Euclidean distance, the Pearson correlation coefficient was also used to measure cell-to-cell methylation distance and has been combined with the HC algorithm to generate cell partitions based on the site-level (Hou et al., 2016) or region-level (Pott, 2017) methylation. Hui et al. developed PDclust to identify cell types using a pairwise dissimilarity (PD) measure and HC, where the PD value was defined as the average of the absolute difference in methylation status at overlapping CpGs between cell pairs (Hui et al., 2018). Despite the considerable diversity in these clustering methods, different (dis)similarity measures could have a significant effect on the quality of the clustering results in distance-based clustering algorithms and no single measure was appropriate for all situations (Yona et al., 2006; Khalifa et al., 2009; Shirkhorshidi et al., 2015). Moreover, only PDclust was verified across different datasets while the clustering performances of other distance measures on different datasets have not been fully evaluated. Recently, a probabilistic hierarchical mixture model Epiclomal was proposed to cluster cells through pooling information across cells and neighboring CpGs (de Souza et al., 2020). But Epiclomal required several non-probabilistic methods for clustering initialization and failed to consistently achieve clustering performance gains than single-distance-based methods on some real datasets. Additionally, Kapourani et al. (2021) proposed the Bayesian models for single-cell methylation data analysis but focused on their evaluation on missing data imputation (Kapourani and Sanguinetti, 2019) and identifying variable features. In summary, additional clustering methodologies that are universal to different kinds of single-cell methylation datasets are still urgently needed.

Recent advancements in ensemble clustering (Ghaemi et al., 2009; Vega-Pons and Ruiz-Shulcloper, 2011; Boongoen and Iam-On, 2018) have demonstrated that integrating various basic cell partitions in a consensus matrix is effective to generate improved clustering solutions (Kiselev et al., 2017; Zhu et al., 2020; Cui et al., 2021; Wang et al., 2021). The rationale for this idea is to construct a cell-to-cell pairwise similarity matrix based on the diverse basic clustering results through a cluster-based similarity partitioning algorithm (CSPA) (Strehl and Ghosh, 2002), with each value in the matrix representing the probability of the occurrence of cell pairs in the same cluster. Then the resulting ensemble cell clusters can be yielded according to the consensus matrix with typical clustering algorithms, such as HC. Since how to accurately capture intercellular methylation (dis)similarity relationships is significant for clustering cells, combining information from multiple (dis)similarity measures to reconstruct the cell-to-cell similarity with the consensus-based clustering strategy becomes a promising alternative. However, the traditional consensus strategy only integrated the information of basic clustering assignments (Golalipour et al., 2021; Zhang, 2021), which might be not sufficiently informative to reconstruct the cell-to-cell similarity as the inherent distance relationships within the subpopulation were ignored. Moreover, when calculating the consensus matrix, the basic clustering partitions could be highly correlated or differ significantly and their ability to distinguish cells was different, requiring an extra strategy to balance the diversity and separability of the basic clustering partitions. Although many weighting strategies based on various clustering validation indices have been proposed to construct a more accurate consensus matrix (Vega-Pons et al., 2008; Vega-Pons et al., 2011; Ünlü and Xanthopoulos, 2019; Zhu et al., 2020), they did not take into account the diversity and separability of basic cluster partitions simultaneously.

Here, we propose scMelody, an enhanced consensus-based clustering model for single-cell methylation data analysis by reconstructing cell-to-cell pairwise similarity. By introducing a regularization process and a dual weighting strategy, scMelody improves the construction of the consensus matrix which contributes to a novel cell-to-cell similarity measure for clustering cells. Compared to the single (dis)similarity measures, the reconstructed cell-to-cell similarity measure combines the multiple inherent distance relationships of cells and the clustering information of basic cell clusters, so as to improve the accuracy of identifying cell subpopulations. As an additional benefit, scMelody can conveniently leverage the internal clustering validation criterion to determine the optimal number of clusters based on the reconstructed pairwise similarity patterns. Extensive assessments on both real datasets and synthetic datasets showed that scMelody achieved the most advanced performance over previous methods in clustering single-cell methylation data.

## 2 Materials and Methods

### 2.1 Datasets and Pre-Processing

We first retrieved seven real single-cell methylation datasets in which cell types were known a priori or were validated in the respective study to benchmark the performance of the clustering algorithms. These distinct single-cell methylation datasets were generated by various sequencing techniques and came from Smallwood et al. (2014), Farlik et al. (2015), Hou et al. (2016), Pott (2017), Farlik et al. (2016) and Luo et al. (2017). The Smallwood dataset was made up of mouse embryonic stem cells (ESCs), where the cells were cultured in a regular serum medium and 2i medium to introduce differential methylation. Note that there were two outlier cells from the serum condition that were demonstrated to be more similar to the 2i ESCs. The Falik2015 dataset consisted of K562 cells and HL60 cells, which were either treated with extra drugs or not, leading to 4 different cell subpopulations. The Hou dataset consisted of the cells were from a human hepatocellular carcinoma (HCC) tissue sample and a human hepatoblastoma-derived cell line (HepG2). There were two subpopulations in HCC cells, where the authors integrated gene expression, copy number changes and DNA methylation to support their findings. The Pott dataset consisted of GM12878 cells and K562 cells, which were grown in different culture mediums. The Farlik2016 dataset contained several different types of human hematopoietic cells, including hematopoietic stem cells (HSC), multipotent progenitors (MPP), common lymphoid progenitor (CLP), common myeloid progenitor (CMP), immature multi-lymphoid progenitor (MLP0), and granulocyte-macrophage progenitor (GMP). The Luo dataset was relatively large, which consisted of two different parts, including 2740 human neurons (Luo-human) and 3,377 mouse neurons (Luo-mouse). According to the original experiment, both the human and mouse neurons were very heterogeneous, where there were 21 subclusters identified in human neurons and 16 subclusters identified in mouse neurons. The overview of these real datasets is summarized in Table 1, including the number of cells and the number of clusters for each dataset. Moreover, in addition to the aforementioned datasets for the standard validation, we also retrieved one of the largest publicly available datasets, which assayed 28077 inhibitory neurons from different regions of the mouse brain and presented strong cellular heterogeneity (Liu et al., 2021). We focused on the evaluation of the ability of scMelody to identify novel cell clusters under complex cell composition contexts on this large dataset.

**TABLE 1:** Overview of the seven real single-cell methylation datasets.

Datasets	Sequencing	# GEO accession	# Cells	# Clusters
Smallwood	ScBS	GSE56879	32	2
Farlik2015	scWGBS	GSE65196	69	4
Hou	scTrio-seq	GSE65364	31	3
Pott	scNOMe-seq	GSE83882	23	2
Farlik2016	scWGBS	GSE87197	122	6
Luo-human	snmC-seq	GSE97179	2740	21
Luo-mouse	snmC-seq	GSE97179	3377	16

To faithfully simulate methylation data that resemble scME-seq for evaluating the clustering stability and scalability of scMelody, we also generated synthetic datasets with various initial settings using the sub-sampling strategy proposed by Kapourani and Sanguinetti (2019). To retain the structure of missing data observed in sequencing experiments, this strategy generated the pseudo-single cells by sampling the raw FASTQ files of the bulk data. We collected the bulk RRBS data (GEO accession: GSE27584) of 10 cell lines (Supplementary Table S1) from the ENCODE dataset (Wang et al., 2012) and the pseudo-single cells were produced by randomly keeping 10% of the mapped reads from the bulk experiment. Then, we generated the synthetic datasets with different initial settings: (1) the number of pseudo-single cells ( N=50,100,200,300,400,500,600,800,1000 ); (2) the number of predefined clusters ( C=2, 3, 4, 5, 6, 7, 8, 9, 10 ); (3) the dropout CpG proportions ( η=0.1, 0.2, 0.3, 0.4, 0.5, 0.6, 0.7, 0.8, 0.9 ). Note that the number of predefined clusters was achieved by combining the cells sampled from different cell lines and we sampled the equal numbers of cells in each cell line. The dropout CpG proportion simulated the data with different sparsity by randomly eliminating a certain proportion of CpG sites in pseudo-single cells, where the higher the dropout proportions represented the higher the degree of data sparsity and the greater difficulty of clustering. In comparative studies, we varied one parameter and kept the others fixed. Unless otherwise specified, the fixed parameters were: number of pseudo-single cells 400, number of predefined clusters 6 and the CpG dropout proportion 0.5. For each setting, we generated 50 input datasets to evaluate the clustering performance.

For the retrieved real single-cell methylation datasets, most of the CpG loci assayed exhibited binary methylation status (methylated or unmethylated). Specifically, the CpGs detected by snmC-seq only had methylated or unmethylated status and the CpGs detected by other sequencing techniques predominantly presented either hypermethylation or hypomethylation (Supplementary Figure S1). Considering the bimodal distribution of methylation levels, the CpGs exhibiting partially methylated calls (≥.5) were assigned a value of 1 (methylation) or a value of 0 (unmethylation) otherwise (<.5). Similarly, for the synthetic datasets generated from the RRBS bulk data, the binary methylation status could be obtained by using a threshold of .5 (values no less than .5 were binarized to 1 otherwise to 0).

### 2.2 scMelody Clustering Algorithm

Considering the sparse coverage of scME-seq technology, scMelody leverages all overlapping CpGs between cell pairs to evaluate cell-to-cell similarity patterns. Specifically, scMelody takes files with binary CpG methylation calls across the genome from individual cells as input. To capture different methylation similarity patterns between cell pairs, scMelody utilizes three correlation-based measures, including Cosine, Hamming and Pearson correlation coefficient, which have been reported to be effective for quantifying the similarity relationships of binary data (Haranczyk and Holliday, 2008). Given a series of single-cell methylation data files $X_{i}$ ( i = 1,2 …, n ; n denotes the number of target cells), the Cosine similarity of cell pairs $\left(X_{i},X_{j}\right)$ can be calculated as follows:

$$S_{1}\left(X_{i},X_{j}\right)=\frac{\sum_{t=1}^{m}X_{it}X_{jt}}{\sqrt{\sum_{t=1}^{m}{\left(X_{it}\right)}^{2}\sum_{t=1}^{m}{\left(X_{jt}\right)}^{2}}}$$

where m represents the number of overlapping CpGs shared by cell pairs $\left(X_{i},X_{j}\right)$ and t denotes t -th overlapping CpG between each cell pair $\left(X_{i},X_{j}\right)$ . For any two cells, the more similar the global methylation landscape is, the larger the Cosine correlation coefficient is; and $S_{1}\left(X_{i},X_{j}\right)$ ranges from 0 to 1. Next, scMelody calculates the Hamming similarity for each cell pair $\left(X_{i},X_{j}\right)$ :

$$S_{2}\left(X_{i},X_{j}\right)=\frac{\sum_{t=1}^{m}I\left(X_{it}=X_{jt}\right)}{m}$$

where the indicator function I(.) returns 1 if its argument is true. This can be described as calculating the proportion of CpGs with concordant methylation status between cell pairs, which ranges from 0 to 1. Finally, the Pearson similarity is calculated as follows:

$$S_{3}\left(X_{i},X_{j}\right)=\frac{\sum_{t=1}^{m}\left(X_{it}-\bar{X_{i}}\right)\left(X_{jt}-{\bar{X}}_{j}\right)}{\sqrt{\sum_{t=1}^{m}{\left(X_{it}-\bar{X_{i}}\right)}^{2}}\sqrt{\sum_{t=1}^{m}{\left(X_{jt}-\bar{X_{j}}\right)}^{2}}}$$

where $\bar{X_{i}}$ , $\bar{X_{j}}$ is the mean of $X_{i}$ , $X_{j}$ respectively and the Pearson similarity measures the linear correlation according to the methylation status between the cell pair $\left(X_{i},X_{j}\right)$ , varying from 0 to 1. With the three basic similarity measures, the inherent methylation similarity relationships of cells can be quantified and the cell-to-cell methylation similarity patterns are captured in the corresponding similarity matrices $\left\{S_{\mu }\text{|}\mu =1,2,3\right\}$ .

To reconstruct the cell-to-cell methylation similarity with the consensus-based clustering strategy, scMelody implements spectral clustering (von Luxburg, 2007) to generate basic cell partitions according to the methylation similarity matrices. Spectral clustering does not make strong assumptions on the form of the cluster and is effective for clustering sparse data with only similarity relationships between data points. Given a similarity matrix $S=\left(s_{ij}\right)\in {\mathbb{R}}^{n\times n}$ , where $s_{ij}$ ≥ 0 represents the linkage weights between cell i and cell j , spectral clustering partitions the cells into C clusters through solving the following optimization problem:

$$\begin{array}{c} min\\ L\in {\mathbb{R}}^{n\times C} \end{array}(LL^{T},I_{n}-\tilde{S}(,\;s.t.\;L^{T}L=I_{C}$$

where $\tilde{S}=D^{-1/2}SD^{-1/2}$ and $D=\text{diag}\left(d_{11},d_{22},\ldots ,d_{nn}\right)$ is a diagonal matrix with $d_{ii}=\overset{n}{\underset{j=1}{\sum }}s_{ij}$ . Finally, each row of obtained L is treated as a data point in ${\mathbb{R}}^{C}$ , and is clustered into C groups by k-means. Note that $I_{n}-\tilde{S}$ is called a normalized graph Laplacian. By implementing spectral clustering on the three similarity matrices { $S_{1},S_{2},S_{3}$ }, we can generate a set of basic cell partitions $\Pi =\left\{\pi_{\mu }\text{|}\mu =1,2,3\right\}$ , which can be used as a clustering prior for reconstructing cell-to-cell similarity.

To convert the information of each basic cell partition into the respective cell-to-cell similarity matrix, scMelody constructs a co-occurrence matrix for each basic cluster. In traditional consensus clustering strategy, for each basic clustering assignment $\pi_{\mu }$ in Π , an n×n binary co-occurrence matrix is constructed, which can be denoted as $I_{\mu }$ :

$$I_{\mu }\left(X_{i},X_{j}\right)=\{\begin{array}{cc} 1 & ifC\left(X_{i}\right)=C\left(X_{j}\right)\\ 0 & otherwise \end{array}$$

where $C\left(X_{i}\right)$ denotes the clustering label of cell $X_{i}$ , and if the cell pairs $\left(X_{i},X_{j}\right)$ are assigned into the same cluster in the μ -th member $\pi_{\mu }$ , the value of $I_{\mu }\left(X_{i},X_{j}\right)$ is equal to 1, otherwise is 0. The general consensus matrix is obtained by averaging the binary co-occurrence matrices $I_{\mu }$ . However, this may not be sufficiently informative to reconstruct cell-to-cell similarity as the inherent similarity relationships of cells are ignored and the resulting consensus matrix is heavily dependent on the basic cell partitions.

To reconstruct the cell-to-cell similarity patterns that faithfully reflects the methylation difference between cells, scMelody adopts a two-stage strategy to improve the construction of the consensus matrix and the resulting consensus matrix can be used to measure the cell-to-cell pairwise similarity in higher resolution. In the first stage, scMelody redefines the construction of the binary co-occurrence matrix $I_{\mu }$ to produce a more fine-grained co-occurrence matrix $I_{\mu }^{*}$ . Specifically, scMelody utilizes the basic similarity matrix to regularize the binary co-occurrence matrix $I_{\mu }$ and the new co-occurrence matrix $I_{\mu }^{*}$ can be expressed as:

$$I_{\mu }^{*}=I_{\mu }\;⊙\;S_{\mu }$$

where ⊙ denotes the Hadamard product and each value in $I_{\mu }^{*}$ can be calculated as $I_{\mu }^{*}\left(X_{i},X_{j}\right)=I_{\mu }\left(X_{i},X_{j}\right)\times S_{\mu }\left(X_{i},X_{j}\right)$ . In this way, the new matrix $I_{\mu }^{*}$ measures the co-occurrence of cell pairs belonging to the same cluster in higher resolution. Compared to $I_{\mu }$ , $I_{\mu }^{*}$ refines the similarity of cells within the clusters, while preserving the differences between cells belonging to different clusters. In the second stage, scMelody adaptively assigns weights to different $I_{\mu }^{*}$ based on the diversity and separability of the basic cell partitions with a dual weighting strategy. Firstly, existing studies have underlined the importance of diversity in basic clustering partitions to enhance the performance of ensemble solutions (Kuncheva and Hadjitodorov, 2004; Hadjitodorov et al., 2006; Fern et al., 2008), thus scMelody proposes a weighting criterion to assess the diversity of basic cell partitions based on NMI (Vinh et al., 2010), where NMI utilizes mutual information to measure the agreement of the two clustering assignments. Suppose each basic cell partition $\pi_{\mu }=\left\{C_{1}^{\mu },C_{2}^{\mu },\ldots C_{k}^{\mu },\ldots ,C_{K\mu }^{\mu }\right\}$ , $C_{k}^{\mu }$ is a cluster of $\pi_{\mu }$ and Kμ denotes the number of the clusters of $\pi_{\mu }$ . To punish the basic cell partition that contributes little to the diversity, the weight for basic cell partition $\pi_{\mu }$ can be formularized as follows:

$$w_{\mu }^{div}=\frac{exp\left(-\frac{1}{r-1}\sum_{\nu =1,\nu \neq \mu }^{r}NMI\left(\pi_{\mu },\pi_{\nu }\right)\right)}{\sum_{\mu =1}^{r}exp\left(-\frac{1}{r-1}\sum_{\nu =1,\nu \neq \mu }^{r}NMI\left(\pi_{\mu },\pi_{\nu }\right)\right)}$$

$$NMI\left(\pi_{\mu },\pi_{\nu }\right)=\frac{2\times \sum_{k,l}p_{kl}log\frac{p_{kl}}{p_{k}\times p_{l}}}{-\sum_{k}p_{k}logp_{k}-\sum_{l}p_{l}logp_{l}}$$

where r=3 represents the number of basic cell partitions. Besides, $p_{k}=n_{k}/n$ , $p_{l}=n_{l}/n$ and $p_{kl}=n_{kl}/n$ , where $n_{k}$ , $n_{l}$ represents the number of cells in the k -th and l -th cluster of the basic cell partition $\pi_{\mu },\pi_{\nu }$ respectively, and $n_{kl}$ is the number of cells shared by cluster k and cluster l . NMI score ranges from 0 to 1, with higher NMI score representing more consistent basic cell partitions and $\frac{1}{r-1}\overset{r}{\underset{\nu =1,\nu \neq \mu }{\sum }}NMI\left(\pi_{\mu },\pi_{\nu }\right)$ measures the overall consistency between the basic cell partition $\pi_{\mu }$ and others, with higher values representing less contribution to the diversity. Note that $0<w_{\mu }^{div}<1$ and $\underset{\mu }{\sum }w_{\mu }^{div}=1$ . Then, to assess the separability of basic cell partitions, scMelody considers the silhouette coefficient (Rousseeuw, 1987), which combines the cohesion and separation of clusters to assess the clustering performance when the ground truth labels are not known. Given a basic cell clustering assignment $\pi_{\mu }=\left\{C_{1}^{\mu },C_{2}^{\mu },\ldots C_{k}^{\mu },\ldots ,C_{K\mu }^{\mu }\right\}$ , the weight defined by the separability can be obtained as follows:

$$w_{\mu }^{sep}=\frac{exp\left(SI\left(\pi_{\mu }\right)\right)}{\sum_{\mu =1}^{r}exp\left(SI\left(\pi_{\mu }\right)\right)}$$

$$SI\left(\pi_{\mu }\right)=\frac{1}{K\mu }\underset{k}{\sum }\left\{\frac{1}{n_{k}}\underset{X_{i}\in C_{k}^{\mu }}{\sum }\frac{b\left(X_{i}\right)-a\left(X_{i}\right)}{\text{max}\left[b\left(X_{i}\right),a\left(X_{i}\right)\right]}\right\}$$

$$a\left(X_{i}\right)=\frac{1}{n_{k}-1}\underset{X_{j}\in C_{k}^{\mu },\;X_{j}\neq X_{i}}{\sum }K\left(S_{\mu }\left(X_{i},X_{j}\right)\right)$$

$$b\left(X_{i}\right)=min_{l,l\neq k}\left\{\frac{1}{n_{l}}\underset{X_{j}\in C_{l}^{\mu }}{\sum }K\left(S_{\mu }\left(X_{i},X_{j}\right)\right)\right\}$$

where $a\left(X_{i}\right)$ denotes the average distance between cell $X_{i}$ and all other cells in the same cluster $C_{k}^{\mu }$ while $b\left(X_{i}\right)$ denotes the average distance between cell $X_{i}$ and all other cells in the next nearest cluster $C_{l}^{\mu }$ . Here, K(.) is a kernel function that converts the similarity measure $S_{\mu }\left(X_{i},X_{j}\right)$ to the respective distance measure $1-S_{\mu }\left(X_{i},X_{j}\right)$ as the original value of the basic cell-to-cell similarity measure varies from 0 to 1. $SI\left(\pi_{\mu }\right)$ ranges from -1 to 1, with a higher value indicating that the intra-class distance is small while the inter-class distance is large thus the cells are well-clustered. Note that we also have $0<w_{\mu }^{sep}<1$ and $\underset{\mu }{\sum }w_{\mu }^{sep}=1$ , with higher $w_{\mu }^{sep}$ indicating higher separability for basic cell partition $\pi_{\mu }$ . In this way, scMelody achieves the assessment of weights based on the diversity and separability of the basic cell partitions. Combining with the regularized co-occurrence matrix $I_{\mu }^{*}$ , the resulting weighted consensus matrix CO can be constructed through a linear aggregation function, which can be expressed as:

$$CO\left(X_{i},X_{j}\right)=f\left(w,I^{*}\right)=0.5*\left(\underset{\mu }{\sum }w_{\mu }^{div}I_{\mu }^{*}+\underset{\mu }{\sum }w_{\mu }^{sep}I_{\mu }^{*}\right)$$

where 0.5 is used as a scaling coefficient, restricting the value of cell-to-cell pairwise similarity in the weighted consensus matrix CO varying from 0∼1. Each value $CO\left(X_{i},X_{j}\right)$ in the resulting weighted consensus matrix is a reconstructed similarity measure of each cell pair $\left(X_{i},X_{j}\right)$ , which measures the methylation similarity relationships between cells in higher resolution.

**FIGURE 1:**
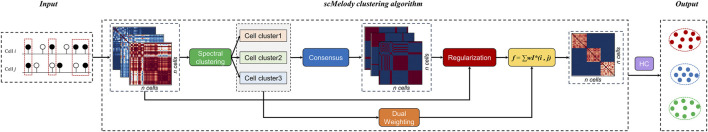
Illustrative flowchart of scMelody. scMelody first utilizes three correlation-based measures to capture cell-to-cell methylation similarity patterns, including Cosine, Hamming and Pearson. The basic cell clusters are generated by spectral clustering according to the similarity patterns. Then, scMelody leverages an enhanced consensus-based clustering model to reconstruct the cell-to-cell similarity by integrating the basic cell clusters and similarity patterns. The resulting cell cluster is generated by performing the complete-linkage hierarchical clustering according to the reconstructed cell-to-cell similarity matrix.

Finally, the weighted consensus matrix CO is clustered using the complete-linkage HC algorithm to yield the resulting cell partitions. The overall scMelody clustering framework is shown in Figure 1, and the pseudo code flow is available in **Algorithm 1**.

Algorithm 1: scMelody

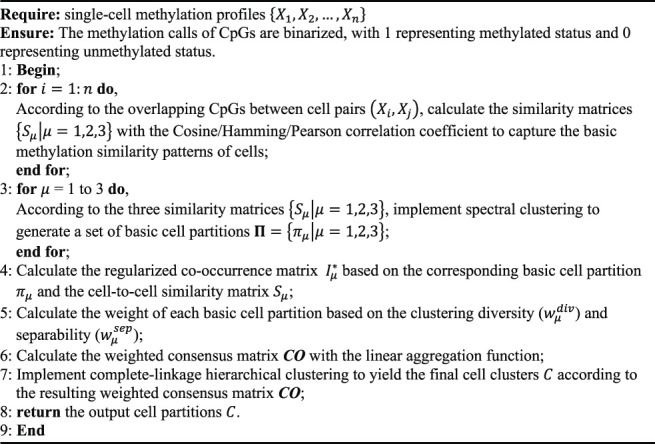


### 2.3 Determine the Optimal Number of Clusters

Both the spectral clustering and HC algorithms need to specify the number of clusters in advance to generate the cluster assignments. Here, we integrate basic similarity measures of cells to propose a robust strategy to determine the optimal number of clusters based on the silhouette coefficient criterion. Let $k=\left\{2,\ldots ,K_{max}\right\}$ , where $K_{max}$ denotes the possible maximum number of clusters, we first run the spectral clustering varying k ( k denotes the input number of clusters for spectral clustering) from 2 to $K_{max}$ . Let $\pi_{k}$ represents the corresponding cell partition when the input number of clusters equaling k . For the three different similarity measures, we can get three different cell partitions at each value of k . Then, we calculate the silhouette coefficient for each similarity measure at each k and select the best $k_{sp}$ as the optimal number of spectral clustering which is given by:

$$k_{sp}=argmax\overset{r}{\underset{\mu =1}{\sum }}\left(SI\left(\pi_{k}\right)\text{|}k\right)$$

where r=3 represents the number of spectral clustering partitions and $\left(SI\left(\pi_{k}\right)\text{|}k\right)$ represents silhouette coefficient of the corresponding spectral clustering partition based on similarity measure μ at each k . $k_{sp}$ is selected as the optimal number for spectral clustering when the sum of the corresponding silhouette coefficients generated from the three basic similarity measures reaches maximum. Then, to generate the final cell partitions, the reconstructed similarity matrix ***CO*** is clustered using the complete-linkage HC algorithm. We cut the hierarchical tree at $k_{opt}$ clusters which can be expressed as:

$$k_{opt}=argmax\left(SI\left(\pi_{k}\right)\text{|}k\right)\;$$

where $k_{opt}$ is the optimal number of the resulting cell partitions and can be obtained when the silhouette coefficient generated from the reconstructed similarity measures reaches maximum.

### 2.4 Model Comparison

To evaluate the clustering performance of scMelody, we performed intensive comparative studies with previously published methods, which were described as follows:

SW + HC (Smallwood et al., 2014): The sliding window (SW) approach first estimated the sample-specific methylation rates of the genome-wide CpGs in a single cell based on a binomial distribution. To increase the coverage across cells, a sliding window of 3 kb in size and 600 bp in step size was used to subdivide the genome. Then the cell-to-cell methylation variances were evaluated using the estimated sample-specific methylation rates. The cell partitions were generated by the complete-linkage hierarchical clustering.

PearsonHC (Hou et al., 2016): This approach utilized the Pearson correlation coefficient to measure cell-to-cell methylation similarity based on the genome-wide overlapping CpGs of cell pairs. This measure was identical to the Pearson similarity metric used in scMelody. The complete-linkage HC was implemented to generate the cell clusters.

PDclust (Hui et al., 2018): PDclust depended on a measure of CpG methylation pairwise dissimilarity (PD), which was defined as the proportion of the overlapping CpGs with discordant methylation status between each pair of cells. The cell partitions were generated by calculating Euclidean distances between each pair of cells based on their PD values using the Ward-linkage HC. Note that the PD value used in PDclust is different from the Hamming similarity measure in scMelody, as the Hamming similarity measure quantified the methylation similarity of cell pairs and the basic cell partitions were obtained based entirely on Hamming similarity without calculating the Euclidean distances of the measure.

Epiclomal (de Souza et al., 2020): Epiclomal was a probabilistic clustering method arising from a hierarchical mixture model which performed better than single-distance-based methods on several datasets. There were two major variants for Epiclomal, including EpiclomalBasic (EpiclomalB) and EpiclomalRegion (EpiclomalR). EpiclomalB considered the methylation status of all CpGs while EpiclomalR focused on the methylation levels across genomic functional regions such as CGIs, leading to better interpretation of the expected cellular heterogeneity on real datasets. Thus, the author mainly focused on the clustering performance of EpiclomalR on real datasets. To be fair, we applied the two versions of Epiclomal on the synthetic datasets; while on the real datasets, only EpiclomalR was considered. For EpiclomalR, the clustering assignments were generated from the filtered inputs of 10,000 CpGs, which were based on the functional genomic regions from CGI and TFBS.

### 2.5 Clustering Performance Metrics

To evaluate the performance of different clustering algorithms, we utilize two popular clustering validation indices, including the Adjusted Rand Index (ARI) (Hubert and Arabie, 1985) and V-measure (Rosenberg and Hirschberg, 2007). Both the two clustering validation indices measure the agreement between the inferred cell clusters and the true or predefined ones from different perspectives. ARI measures clustering performance by the similarity or matching degree between the prediction target cluster vector and the real cluster vector. Given a set of m cells, the quantitative relationship between the clustering results and the reference labels can be reflected in a contingency table, where each entry indicates the number of objects in common between the prediction and the reference.

$$ARI=\frac{\sum_{ij}\left(\begin{array}{c} m_{ij}\\ 2 \end{array}\right)-\left[\sum_{i}\left(\begin{array}{c} \alpha_{i}\\ 2 \end{array}\right)\sum_{j}\left(\begin{array}{c} \beta_{j}\\ 2 \end{array}\right)\right]/\left(\begin{array}{c} m\\ 2 \end{array}\right)}{\frac{1}{2}\left[\sum_{i}\left(\begin{array}{c} \alpha_{i}\\ 2 \end{array}\right)+\sum_{i}\left(\begin{array}{c} \beta_{j}\\ 2 \end{array}\right)\right]-\left[\sum_{i}\left(\begin{array}{c} \alpha_{i}\\ 2 \end{array}\right)\sum_{j}\left(\begin{array}{c} \beta_{j}\\ 2 \end{array}\right)\right]/\left(\begin{array}{c} m\\ 2 \end{array}\right)}$$

Where $m_{ij}$ comes from the contingency table, $\alpha_{i}$ is the sum of the $i^{th}$ row of the contingency table, $\beta_{j}$ is the sum of the $j^{th}$ column of the contingency table and the (.) function denotes a binomial coefficient. The V-measure captures the homogeneity and completeness of a clustering result. To satisfy the homogeneity criterion, each cluster contains only members of a single class. Completeness is satisfied if all those cells that are members of a single group are assigned to a single cluster. The V-measure can be calculated as the harmonic mean of homogeneity ( h ) and completeness ( c ):

$$V=\frac{2hc}{h+c}$$

where the homogeneity h=1−H(C|K)/H(C) , H(C|K) is the conditional entropy of the classes given the cluster assignments and is given by $H\left(C\text{|}K\right)=-\overset{\left|C\right|}{\underset{c=1}{\sum }}\overset{\left|K\right|}{\underset{k=1}{\sum }}\frac{n_{c,k}}{n}log\left(\frac{n_{c,k}}{n}\right)$ , H(C) is the entropy of the classes and is given by $H\left(C\right)=-\overset{\left|C\right|}{\underset{c=1}{\sum }}\frac{n_{c}}{n}log\left(\frac{n_{c}}{n}\right)$ , with n the number of cells, $n_{c}$ and $n_{k}$ the number of cells respectively belonging to true class c and cluster k , and $n_{c,k}$ the number of cells from true class c assigned to cluster k . The completeness c=1−H(K|C)/H(K) , which has the analogous formulation as the homogeneity h .

## 3 Results

### 3.1 scMelody Outperforms the Existing Methods

**FIGURE 2:**
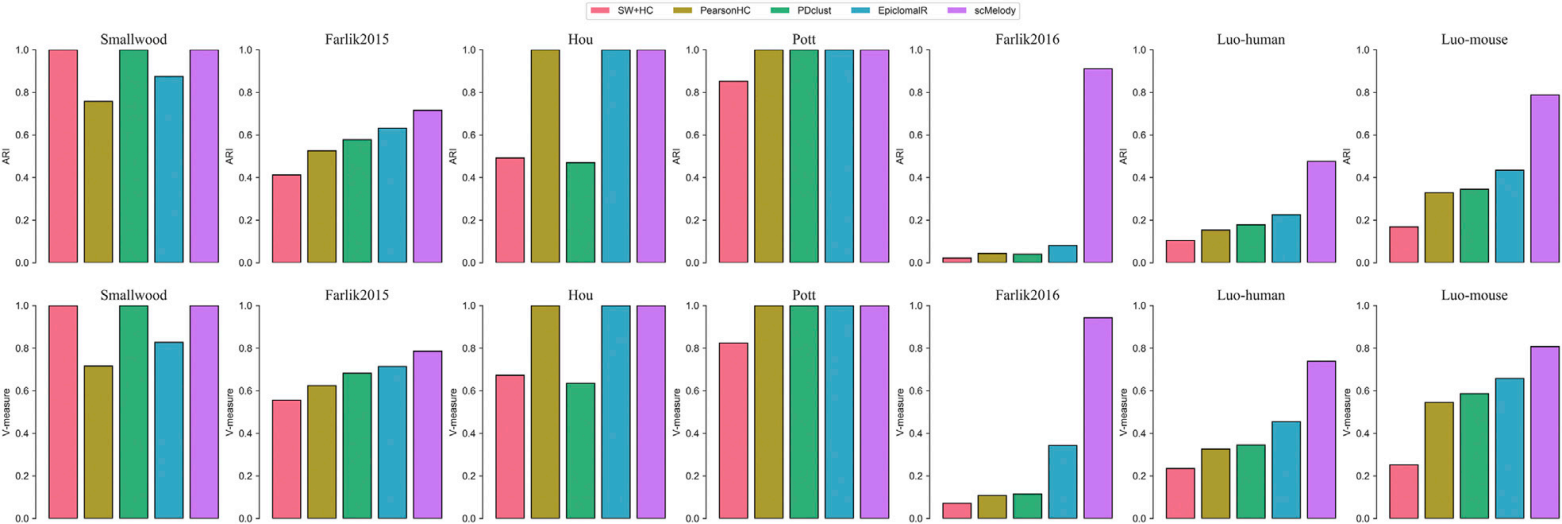
Clustering performance comparison between scMelody and other major published methods on the real datasets. Both ARI and V-measure are employed to assess the similarity between inferred and true cluster labels.

We first benchmarked scMelody together with the other published methods on 7 real single-cell methylation datasets, reflecting a wide spectrum of sequencing techniques, data sparsity, number and heterogeneity of single cells examined. Figure 2 showed the clustering performance of these methods across the datasets, which clearly indicated that scMelody outperformed other methods by achieving the same or higher ARI and V-measures scores. Specifically, on the three datasets with fewer cells or clusters, including Smallwood, Hou and Pott, scMelody accurately clustered all cells while other methods misclassified one or several cells. On the Farlik2015 dataset, the heterogeneity between the different cell subpopulations (treated or untreated) was subtle, however, scMelody performed better than the competing methods by achieving less misclassification for both K562 and HL60 treated cells. On the Farlik2016 dataset, scMelody achieved significant clustering performance gains than other methods, where the inferred assignments of scMelody showed much higher consistency with the true cell clusters (Supplementary Figure S2). On the two relatively large datasets, scMelody was superior to the competing methods by recapitulating the major cell types more accurately and achieved higher ARI and V-measure scores. Moreover, EpiclomalR accurately identified the cell heterogeneity on both Hou and Pott datasets and was slightly inferior to scMelody on Smallwood and Farlik2015 datasets while was significantly inferior to scMelody on Farlik 2016, Luo-human and Luo-mouse datasets. The clustering performances of the three single-distance-based methods varied a lot across different datasets. On the simple datasets with fewer numbers of cells or clusters (like Smallwood and Pott), they could accurately identify the cell heterogeneity and achieved close ARI or V-measure scores compared to scMeldoy and EpiclomalR; however, their clustering performance decayed rapidly on complex datasets with increasing numbers of cells or clusters (like Farlik2016 and Luo-human). Additionally, we also observed that even the three single-distance-based methods achieved different clustering performances on different datasets and no single measure could always be better than others. Supplementary Figure S3 summarized the ARI scores and V-measure scores of the benchmarked methods across the real datasets and scMelody showed the highest average ARI and V-measure scores, indicating that our model was universal to different kinds of single-cell methylation datasets.

We further investigated the performance of the benchmarked methods in terms of estimating the number of clusters. Since only EpiclomalR and scMelody provided built-in functions for predicting the number of clusters, we utilized the silhouette coefficient criterion to specify the optimal number of clusters for the three single-distance-based methods. The result showed that all methods accurately estimated the optimal number of clusters on the datasets with the fewer true numbers of clusters, including the Smallwood and Pott datasets (Table 2). While on the datasets with stronger cellular heterogeneity, scMelody achieved improved estimations that were closer to the numbers of true clusters, such as accurately predicting the number of clusters on the Farlik2016 and Luo-mouse datasets and achieving smaller prediction errors on the Luo-human dataset. EpiclomalR provided better prediction performance than the three single-distance-based methods while the three single-distance-based methods generally underestimated the number of clusters. Of note, although scMelody and the three single-distance-based methods all predicted the number of clusters based on the silhouette coefficient criterion, the better prediction performance of scMelody suggested that the reconstructed cell-to-cell similarity enabled a more accurate reflection of the differences between cell subpopulations.

**TABLE 2:** The estimated number of clusters on each real dataset.

Datasets	True clusters	SW + HC	PearsonHC	PDclust	EpiclomalR	scMelody
Smallwood	2	2	2	2	2	2
Farlik2015	4	2	2	2	2	2
Hou	3	2	3	3	3	3
Pott	2	2	2	2	2	2
Farlik2016	6	2	3	2	7	6
Luo-human	21	13	14	15	25	18
Luo-mouse	16	10	12	12	15	16

### 3.2 scMelody Defines a Better Similarity Measure With Improved Clustering Performance

To further illustrate that scMelody could improve the clustering performance by reconstructing cell-to-cell similarity with the proposed enhanced consensus clustering strategy, we further investigated the clustering results generated by different similarity measures. Using the HC as the benchmarked clustering algorithm, the cell partitions were generated from different similarity matrices: 1) The three basic similarity matrices, including Cosine, Hamming and Pearson. 2) Consensus-I, the similarity matrix was the traditional consensus matrix generated by averaging the binary co-occurrence matrices without the regularization process and the weighting process. 3) Consensus-II, the similarity matrix was the consensus matrix generated by averaging the regularized co-occurrence matrices without the weighting process. 4) Consensus-III, the similarity matrix was the consensus matrix generated by weighting the binary co-occurrence matrices without the regularization process. 5) The similarity matrix was the resulting consensus matrix of scMelody. The differences between these similarity measures are summarized in Table 3.

**TABLE 3:** The differences between the benchmarked similarity measures.

Similarity	Consensus	Regularization	Weighting
Cosine	No	—	—
Hamming	No	—	—
Pearson	No	—	—
Consensus-I	Yes	No	No
Consensus-II	Yes	Yes	No
Consensus-III	Yes	No	Yes
ScMelody	Yes	Yes	Yes

**FIGURE 3:**
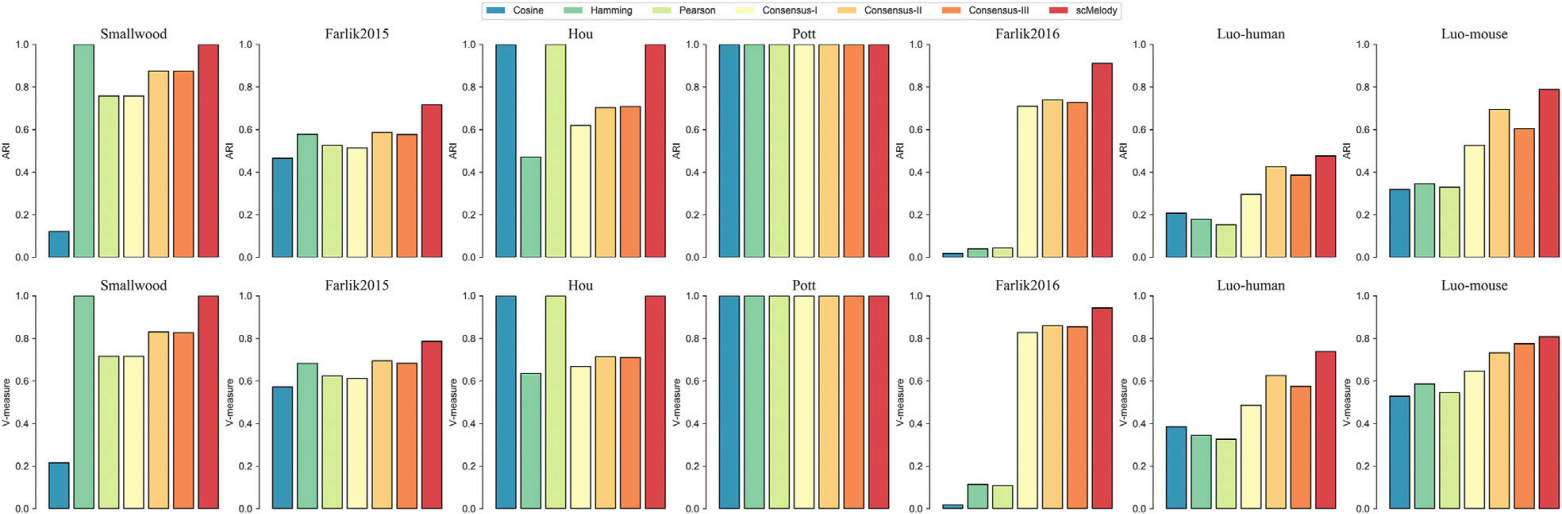
Clustering performance comparison of different similarity measures on the real datasets. These similarity measures include the three basic correlation-based measures and the consensus-based similarity measures. The complete-linkage hierarchical clustering is used as the benchmarked clustering algorithm.

The results showed that the clustering performance varied considerably between different similarity measures (Figure 3). Firstly, we observed that the reconstructed cell-to-cell similarity by scMelody could dissect cellular heterogeneity more accurately and robustly, as it achieved better or the same clustering performance than other similarity measures across all the datasets. Secondly, we also observed that the clustering performances of the basic similarity measures varied considerably on different datasets, indicating that they captured methylation differences between cells from different aspects. Thirdly, generally speaking, integrating the information from basic similarity measures could more accurately reflect the true methylation heterogeneity between cells, which was reflected in the improved clustering accuracy of the consensus-based similarity measures than the basic similarity measures on most datasets. However, we also observed that Consensus-I did not consistently improve the clustering performance on all datasets (like the Smallwood, Farlik2015 and Hou datasets) compared to the basic similarity measures, indicating the limitation of the traditional consensus strategy. Moreover, the overall performance of Consensus-I was not as good as Consensus-II or Consensus-III and this suggested that both the regularization and weighting strategy contributed to boosting the clustering performance. In conclusion, the reconstructed similarity measure by scMelody could achieve more significant clustering performance gains than the basic similarity measures across different real datasets.

### 3.3 Clustering Stability and Scalability of scMelody

**FIGURE 4:**
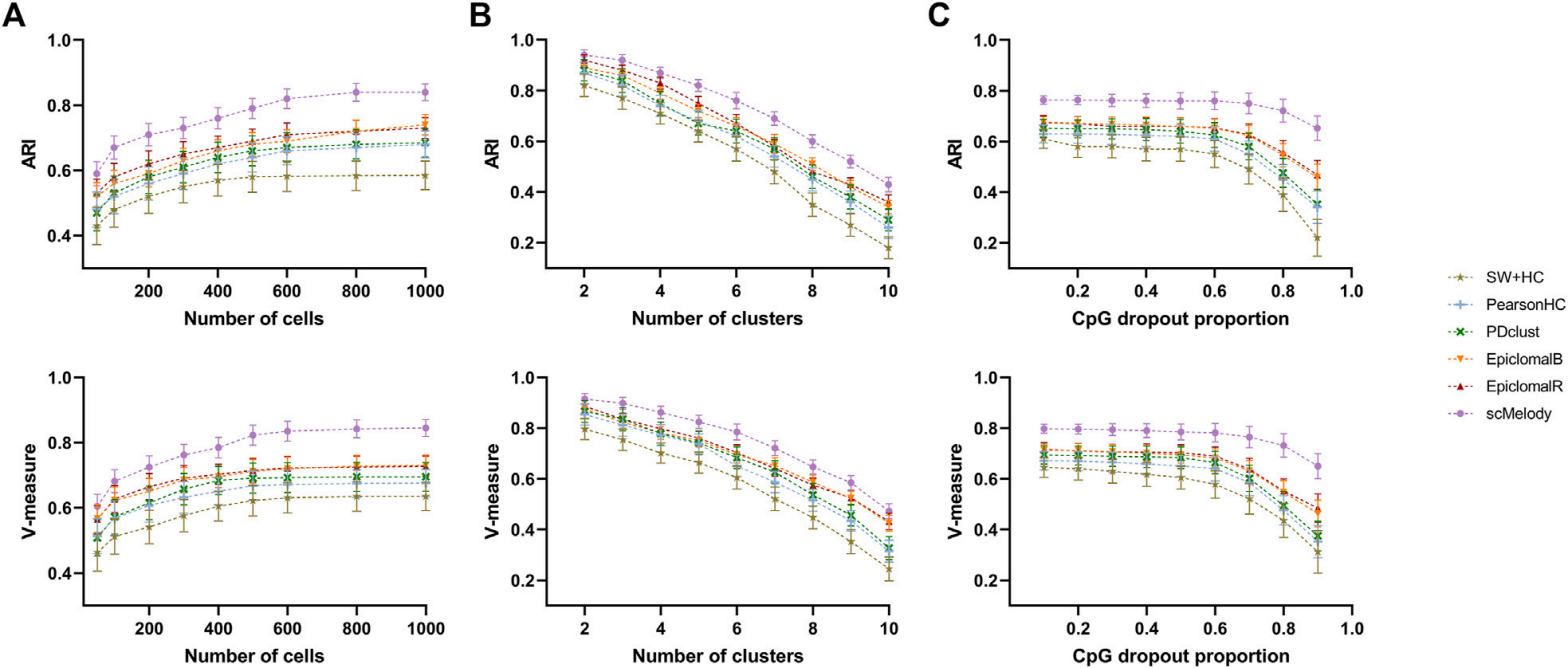
Benchmarking the clustering stability of scMelody and other major published methods on a variety of synthetic datasets. The clustering performance is measured by ARI and V-measure when we vary by: **(A)** number of cells; **(B)** number of clusters; **(C)** CpG dropout proportions. Each setting covers 50 input datasets to evaluate the average clustering performance.

After verifying the clustering performance of scMelody on the real datasets, we generated a variety of synthetic datasets to further evaluate its clustering stability, where the clustering complexity could be controlled with different initialization settings. Firstly, we compared the clustering performance of scMelody and other published methods when the number of cells varied over a wide range. The results showed that when we fixed the number of clusters ( C=6 ) and the CpG dropout proportion ( η=0.5 ), the clustering performance of all methods improved with the increase of the cell numbers, while scMelody performed better than other methods across all settings of cell numbers (Figure 4A). Compared with EpclomalB, EpiclomalR had better average clustering performance when the numbers of cells were small ( N≤600 ), but EpiclomalB outperformed EpiclomalR when the numbers of cells were relatively large, indicating that using the information from genome-wide CpGs might better capture cellular heterogeneity than local functional regions when clustering a large number of cells. We also observed that the two correlation-based methods (PearsonHC and PDclust) were better than the method (SW + HC) based on the Euclidean distance. Figure 4B showed the clustering performance of the benchmarked methods when varying numbers of clusters (with N=600 and η=0.5 ). When the predefined numbers of clusters were small, the differences in clustering performance among the methods were not significant due to the lower complexity of the clustering task; however, with the increase of the number of clusters, the clustering performance of all methods began to drop while scMelody achieved higher average ARI and V-measure scores than the competing methods. Epiclomal performed better than other single-distance-based clustering methods, while PDclust and PearsonHC were better than SW + HC. Finally, when varying the sparsity of the synthetic datasets by CpG dropout proportions, scMelody achieved better clustering performance under all CpG dropout proportions than the competing methods and could maintain the clustering accuracy across a wide range of dropout proportions ( η≤0.7 ), demonstrating its capability and sensitivity in robustly identifying cell subpopulations (Figure 4C).

**FIGURE 5:**
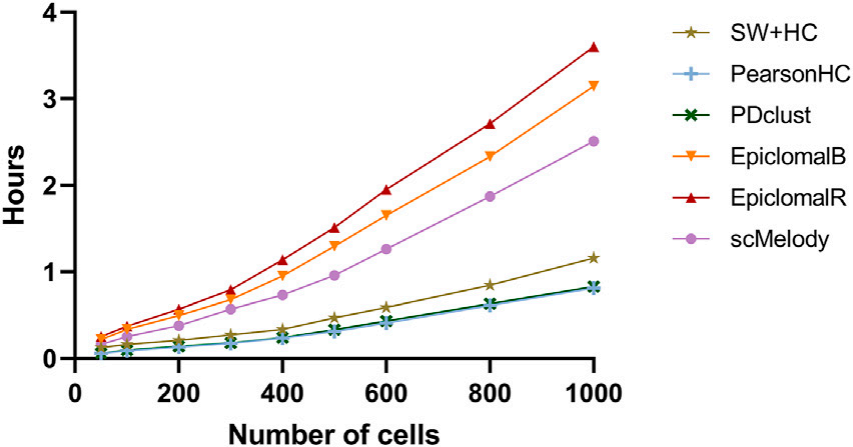
The average runtime of the benchmarked methods on the synthetic datasets with different numbers of cells.

Furthermore, considering that current single-cell methylation sequencing techniques have already assayed tens to thousands of cells, we also evaluated the runtime of these methods at different cell numbers. Note that all calculation was performed on a Windows server with an Intel Xeon Platinum 8160 CPU (2.1 GHz) and 32G RAM. Figure 5 summarized the average time consumption of the benchmarked methods on the synthetic datasets at different numbers of cells. It was obvious that the three single-distance-based methods had lower time consumption than Epiclomal and scMelody, in which SW + HC required more running time than PearsonHC and PDclust. Moreover, scMelody was more computationally efficient compared to EpiclomalB and EpiclomalR while EpiclomalR was more computationally expensive than EpiclomalB. Of note, we found that scMelody spent more than 99% of the running time on calculating the basic cell-to-cell similarity matrices for the input single-cell methylation profiles (Supplementary Figure S4) and this was also true for single-distance-based methods, such as PearsonHC and PDclust. Since scMelody was demonstrated to be stable over a wide range of CpG dropout proportions, researchers were recommended to select CpGs from genomic regions of interest to speed up the calculation of the basic similarity matrices in real application scenarios. Besides, considering the varying number of CpGs assayed in real single-cell methylation datasets, Supplementary Table S2 also showed the runtime of the benchmarked methods on the real datasets and the runtime of scMelody varied within several hours which was practical. To sum up, scMelody could accurately cluster thousands of cells within hours, reaching a balance between the clustering accuracy and the computation efficiency.

### 3.4 The Reconstructed Similarity Facilitates to the Interpretation of Cell Heterogeneity

**FIGURE 6:**
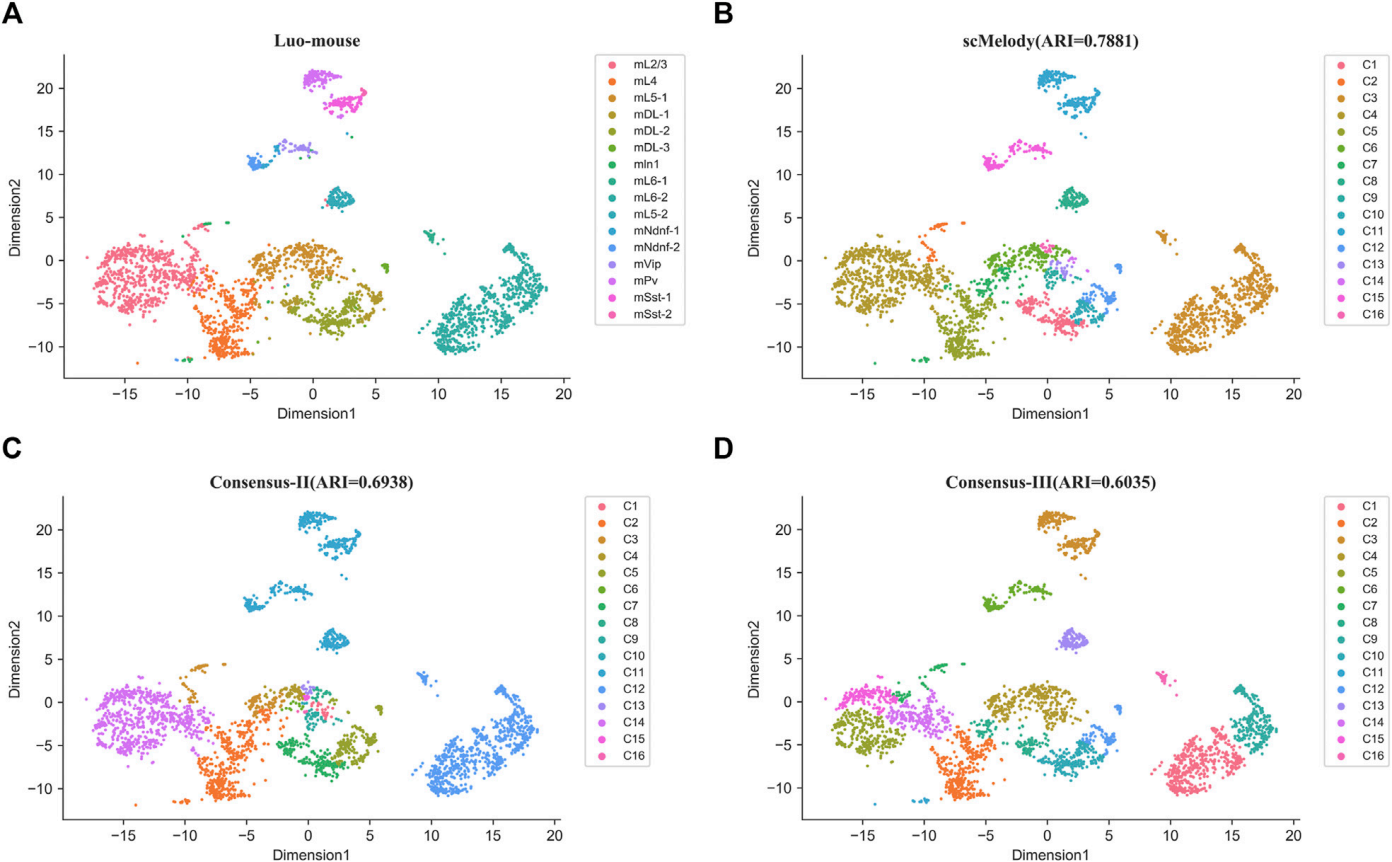
t-SNE visualization results of the Luo-mouse dataset according to the different cell labels. **(A)** True cell labels; **(B)** The inferred clusters of scMeldoy; **(C)** The inferred clusters of Consensus-II. **(D)** The inferred clusters of Consensus-III.

To further demonstrate the ability of scMelody to uncover known cell types, we presented two real case studies for the Smallwood and Luo-mouse datasets. Firstly, we investigated whether the cell-to-cell similarity values could visually assess the structures of cell subpopulations, including the reconstructed similarity measure and the three basic similarity measures. Supplementary Figure S5 showed the heatmaps based on the cell-to-cell pairwise similarity values for the Smallwood dataset. It could be observed that cells with the reconstructed similarity values by scMelody presented a grouping tendency in the diagonal (Supplementary Figure S5A), indicating two significant heterogeneous cell populations on this dataset. Combined with the true cell labels, we found that the two major subpopulations were precisely representative of 2i ESCs and serum ESCs. However, even the basic similarity measures also provided accurate clustering results, like Hamming similarity measure, they could not provide the same aggregation tendency in the diagonal as scMelody did (Supplementary Figures S5B–S5D). This indicated that the reconstructed cell-to-cell similarity could contribute to the characterization of methylation heterogeneity between cells, which could help researchers intuitively assess the potential cell subpopulations. Secondly, we further investigated the clustering results of the consensus-based similarity measures and focused on the effects of the regularization process and the dual weighting strategy on the output cell clusters. Based on the methylation levels in 100 kb bins across the genome, Figure 6 showed the t-SNE(van der Maaten and Hinton, 2008) visualization results of the Luo-mouse dataset according to the original cell types and inferred clusters, where the inferred clusters were generated by different consensus clustering strategies, including scMelody, Consensus-II and Consensus-III (Table 3). The results indicated that scMelody generated more accurate cell clusters which showed a better agreement with the original cell types. Compared to Consensus-II and Consensus-III, scMelody could more accurately identify the major differences between cell subpopulations and avoid overestimating cellular heterogeneity within the subpopulations. This demonstrated the capability of the enhanced consensus-based clustering model to uncover the cell subpopulations, which could boost the clustering performance by integrating the regularization process and the dual weighting strategy.

### 3.5 scMelody Uncovers Novel Cell Clusters

**FIGURE 7:**
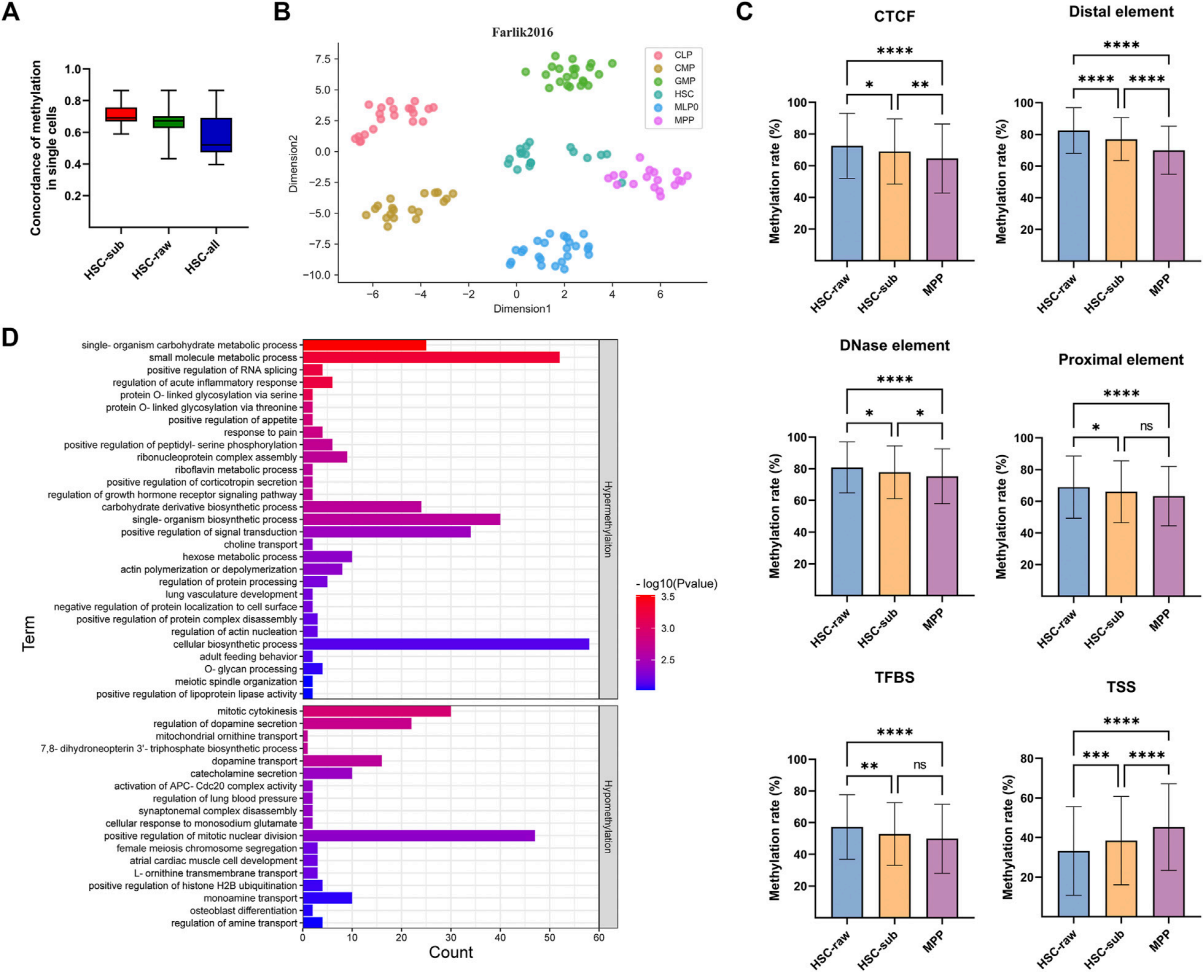
Case study of scMelody in identifying novel cell clusters on the Farlik2016 dataset. **(A)** The concordance of the DNA methylation of the cells annotated as HSC. The concordance is calculated by averaging the pairwise correlation coefficients between any two single cells within each group, including Cosine, Hamming and Pearson correlation coefficient. **(B)** t-SNE projection plot of the Farlik2016 dataset using the average methylation levels on the top 500 variable functional regions in all six types of putative regulatory regions. Each point represents an individual cell, which is colored according to the annotated cell labels from the original experiment. **(C)** Average methylation levels of cells denoted as HSC-raw, HSC-sub and MPP on the six functional genomic regions, including CTCF binding site (CTCF), Distal element, DNase element, Proximal element, Transcription factor binding site (TFBS) and Transcriptional start site (TSS). Tukey’s multiple comparisons test is used to determine whether there is a significant difference in mean methylation levels between each pair of the three cell groups. By default, the significance level is .05 and the significance marks are denoted by: ns, not significant; *p < .05; **p < .01, ***p < .001; ****p < .0001. **(D)** Genomic Regions Enrichment of Annotations Tool (GREAT) enrichment analysis of the variable genomic regions based on biological process Gene Ontology (GO) terms between the HSC-sub and HSC-raw. The enriched GO terms are ordered with the binomial test *p* value.

To demonstrate the capability of scMelody in identifying novel cell clusters, we presented two case studies. Firstly, according to the annotations from the original experiment of the Farlik2016 dataset, the clustering result of scMelody showed that six cells (denoted as HSC-sub) annotated as HSC were clustered as MPP (Supplementary Table S3) while the remaining HSCs (denoted as HSC-raw) were independently grouped together (Supplementary Figure S6). To explore the cause of the deviation, we first examined the pairwise methylation similarity of all cells which were annotated as HSC according to their genome-wide methylation status (Figure 7A). The result showed that cells denoted as HSC-sub or HSC-raw showed high internal correlations and was much higher than assembling them together (HSC-all), indicating potential heterogeneity among the two subpopulations (HSC-sub and HSC-raw). Then, to provide a biologically meaningful basis for analyzing DNA methylation differences between the HSCs and MPPs, we further aggregated the DNA methylation profiles at the functional genomic region level according to the BLUEPRINT version of the Ensembl Regulatory Build (Zerbino et al., 2015; Adams et al., 2012), including six types of putative regulatory regions. Figure 7B showed the t-SNE visualization result of all cells in the Farlik2016 dataset according to their annotated cell labels. We observed that the HSC population was more heterogeneous and a few HSCs presented a closer distance to MPPs. Moreover, Figure 7C showed the average methylation levels of the three groups of cells in the 500 most variable regions (Chi-square, FDR <.05) for each type of the regulatory region. According to Tukey’s multiple comparisons test (Dunn, 1961), the average methylation level of the HSC-sub population was significantly different from that of the HSC-raw population in all six functional regions while was significantly different from that of the MPP population in four of six functional regions. The specific statistic information of the average methylation levels of the three groups of cells could be obtained in Supplementary Tables S4–S9. Moreover, we utilized the GREAT tool (McLean et al., 2010) to evaluate the functional significance of the identified variable genomic regions and the result indicated several enriched biological process (BP) Gene Ontology (GO) terms that were associated with HSC-raw and HSC-sub (Figure 7D; Supplementary Table S10). For instance, the two GO terms mitotic cytokinesis and positive regulation of mitotic nuclear division that were associated with hypomethylation in HSC-raw demonstrated that HSC-raw might have stronger differentiation potency than HSC-sub as DNA methylation could be associated with transcriptional repression (Luo et al., 2018). Finally, combined with the human hematopoietic lineage (Doulatov et al., 2012; Farlik et al., 2016), we knew that all blood cells originated from HSCs and the transition from HSC to MPP was always in the first stage of the differentiation lineage. These findings suggested that the six cells, which were annotated as HSC from the original publication, were different from the typical HSCs and presented an intermediate methylation status of two kinds of continuously differentiated cells (HSC and MPP) that warranted further investigation.

**FIGURE 8:**
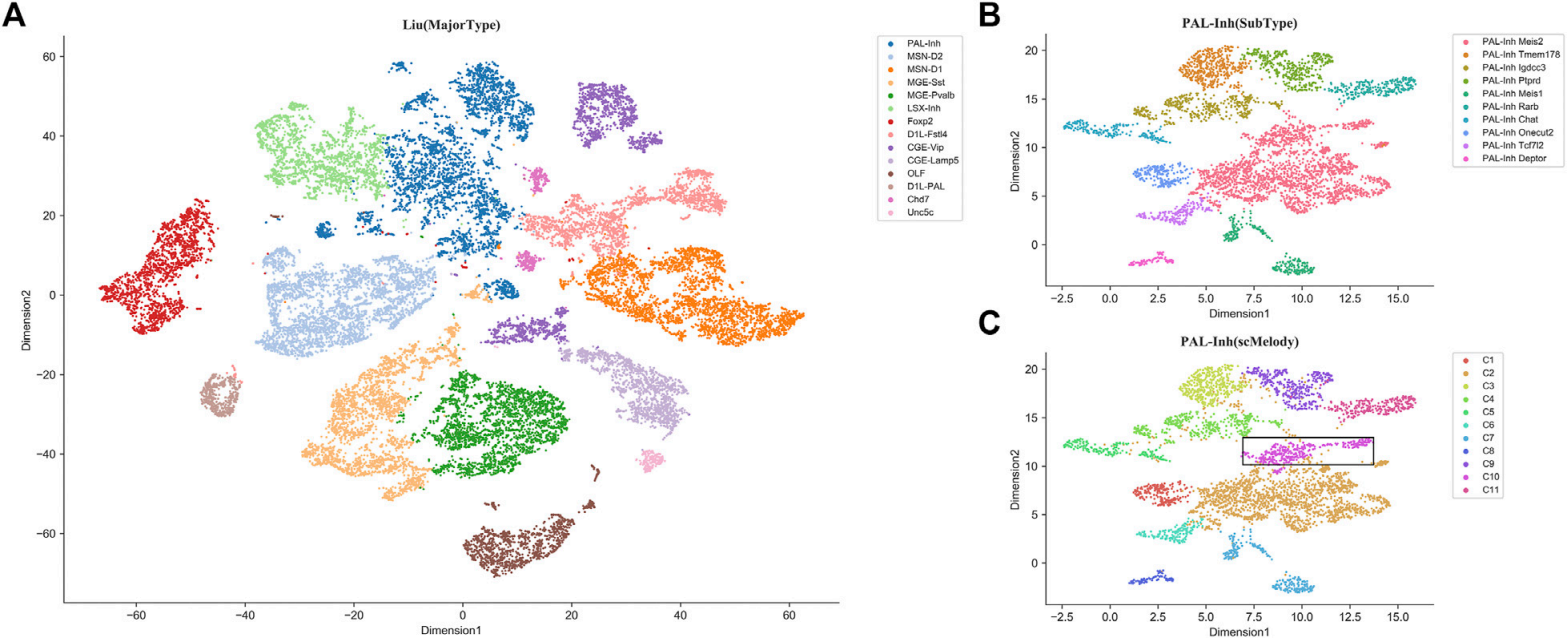
t-SNE visualization results of the large Liu dataset based on the 100 kb bins methylation profiles. **(A)** The t-SNE visualization result of all inhibitory neurons, where a total of 28077 cells are defined as 14 major types and are colored according to the annotations from the original experiment. **(B)** The t-SNE visualization result of PAL-Inh subpopulation, where a total of 4307 cells are defined as 10 subtypes are colored according to the annotations from the original experiment. **(C)** The t-SNE visualization result of PAL-Inh subpopulation, where the cells are clustered into 11 subtypes by scMelody. For comparison, the novel cell cluster identified by scMelody is circled with a black rectangle.

As an additional validation, we also evaluated the ability of scMelody to identify the novel cell clusters on a large dataset with complex cell composition contexts. This dataset was generated by Liu et al. (2021), in which there were 28077 inhibitory neurons derived from different regions of the mouse brain tissue, presenting high intercellular heterogeneity. We first aggregated the methylation profiles of 100 kb bins and these cells could be divided into 14 major types according to the annotations of the original experiment (Figure 8A). Besides, each major type was comprised of multiple heterogeneous subtypes, which were identified in the original experiment. When applying scMelody to this dataset, the clustering results showed that one major type PAL-Inh (inhibitory neurons derived from mouse pallidum) with the largest number of cells (4307 cells) among the 14 major types could be further divided into 11 subtypes, while only 10 subtypes were annotated for the PAL-Inh cells in the original experiment (Figures 8B,C). After comparison, we found that the novel subpopulation (PAL-Inh novel) identified by scMelody mainly came from the subtype PAL-Inh Meis2. Since the methylation levels on gene bodies negatively correlated with the gene expression in mouse neurons (Lister et al., 2013; Mo et al., 2015; Stroud et al., 2017; Liu et al., 2021), we profiled the methylation levels along the gene bodies with Chi-square (FDR <0.05) and the GO analysis revealed enriched BP terms for the differentially methylated genes between the PAL-Inh novel subpopulation and PAL-Inh Meis2 subpopulation (Supplementary Figure S7; Supplementary Table S11). For instance, several most significantly enriched GO terms, such as nervous system development and neurogenesis, clearly showed major biological processes of mouse neuron development. Moreover, we also noticed that the GO term “cell morphogenesis involved in neuron differentiation” was associated with hypermethylation in PAL-Inh novel subpopulation and the GO term “negative regulation of protein modification process” was associated with hypomethylation in PAL-Inh novel subpopulation. This result showed that the PAL-Inh Meis2 subpopulation might have a stronger differentiation ability than the PAL-Inh novel subpopulation (Menon and Gupton, 2018; Badimon et al., 2020). Besides, the GREAT analysis uncovered the term “abnormal neuron morphology” of Mouse Phenotype, which further confirmed the difference in these two cell subpopulations.

## 4 Discussion

The high resolution of single-cell methylation sequencing enables researchers to explore cell-to-cell epigenetic heterogeneity and underlines the significance of clustering cells based on the single-cell methylation profiles. In a biological sense, DNA methylation is well suited for exploring cell heterogeneity because this crucial modification is cell-type-specific and preserves an epigenetic memory of a cell’s developmental history (Farlik et al., 2016). In this paper, we propose scMelody, an enhanced consensus-based clustering model for single-cell methylation data analysis by reconstructing cell-to-cell pairwise similarity. When applying it on real single-cell methylation datasets generated from various sequencing techniques, scMeldoy achieved significant clustering performance gains over the previous methods, including several single-distance-based methods and one probabilistic method. Benefiting from the reconstructed cell-to-cell similarity measure, scMelody also attained accurate estimates for the number of clusters based on the silhouette coefficient criterion. Moreover, using the synthetic datasets generated across a variety of settings, scMelody was demonstrated to be stable which robustly maintained its clustering accuracy over a wide range of number of cells, number of clusters and CpG dropout proportions. The real case studies also indicated the capability of scMelody to identify known cell types and uncover novel cell clusters. To sum up, scMeldoy could accurately recapitulate the cellular epigenetic heterogeneity and was demonstrated to be universal for different kinds of single-cell methylation datasets.

Generally, the (dis)similarity measure is the core for quantifying the methylation differences between cells, thus many methods are designed to incorporate different cell-to-cell methylation (dis)similarity measures into the distance-based clustering algorithms to generate cell partitions. However, our results showed that no single (dis)similarity measure could provide satisfactory clustering performance on all datasets as different (dis)similarity measures captures the cellular heterogeneity from different perspectives. For example, both PearsonHC and PDclust accurately assigned all cells to their respective clusters on the Pott dataset while they could hardly identify the cell types on the Farlik2016 dataset (Figure 4). Instead, a significant advantage of scMelody was that it integrated the clustering information of multiple basic similarity measures to overcome their limitation in capturing complete cellular methylation heterogeneity. Besides, the reconstructed cell-to-cell similarity measure enabled scMelody to reach better clustering performance across different datasets. This highlighted the importance of identifying cell subpopulations by combining the information of different cell-to-cell methylation (dis)similarity relationships. However, even scMelody can process thousands of cells within several hours, the computational efficiency of scMelody is still to be improved especially when the computational resources are limited. We will continue to develop optimized versions of scMelody to improve its computational efficiency, such as the GPU-accelerated scMelody, which can be more practical for the researchers to use it.

With the development of single-cell methylation sequencing technologies, the increase of sequencing depth will greatly alleviate the sparsity problem of single-cell methylation data, which can significantly boost the performance of clustering cells based on cell-to-cell similarity patterns. Our scMelody is flexible and can easily accommodate additional similarity measures to cluster cells, as the novel and sophisticated distance measures continue to be proposed. This has important implications for fully utilizing single-cell methylation sequencing to study cell differentiation versus variation, especially for uncovering novel cell types in complex human diseases, such as cancers.

## Data Availability

An implementation of scMelody is freely available at https://github.com/TQBio/scMelody. All datasets used in this paper can be obtained from the GEO database (https://www.ncbi.nlm.nih.gov/geo/). The synthetic datasets are generated from the bulk RRBS data with GEO accession number GSE27584 and the real single-cell methylation datasets analyzed in this paper can be obtained with the corresponding GEO accession numbers (Table 1). The large Liu dataset can be obtained with GEO accession number GSE132489.
