# Supplementary material for: scMelody: An Enhanced Consensus-Based Clustering Model for Single-Cell Methylation Data by Reconstructing Cell-to-Cell Similarity
Source: Front Bioeng Biotechnol. 2022 Feb 23;10:842019. doi: 10.3389/fbioe.2022.842019 (PMC8905497; doi:10.3389/fbioe.2022.842019)
Supplement: Supplementary file 1 [file DataSheet1.docx]

Supplementary Material

# Supplementary Figures and Tables

## Supplementary Figures


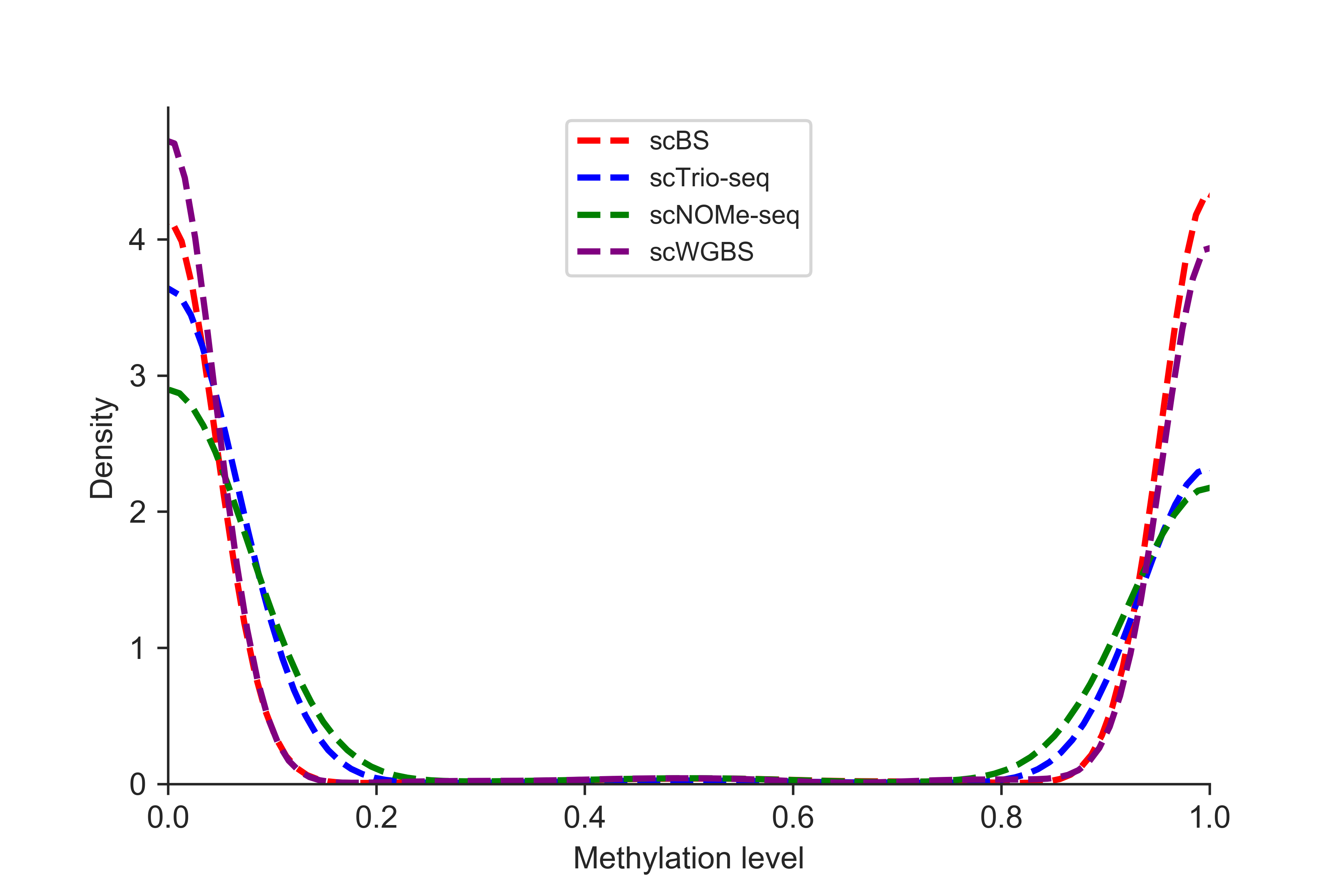


**Supplementary Figure 1.** The distributions of the genome-wide methylation levels for the single-cell methylation data generated by different sequencing techniques.


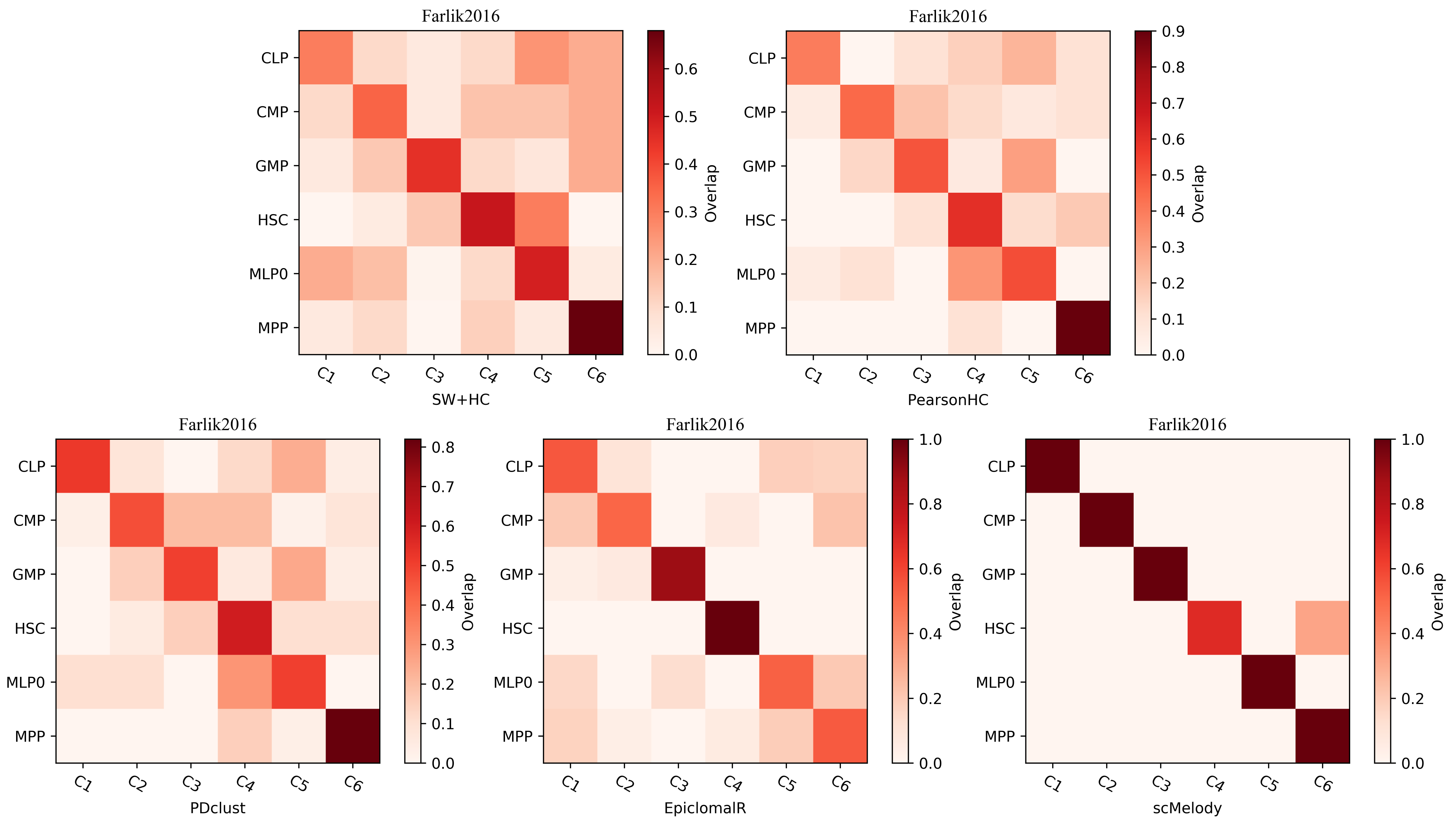


**Supplementary Figure 2.** Co-clustering between the published cell types on the rows and the inferred clusters on the columns for the Farlik2016 dataset. Each entry is the percentage of cells in published class i that are present in predicted cluster j, with the rows summing up to 1. A perfect agreement between the inferred clusters and the published cell types would result in a square matrix with a deep red diagonal.


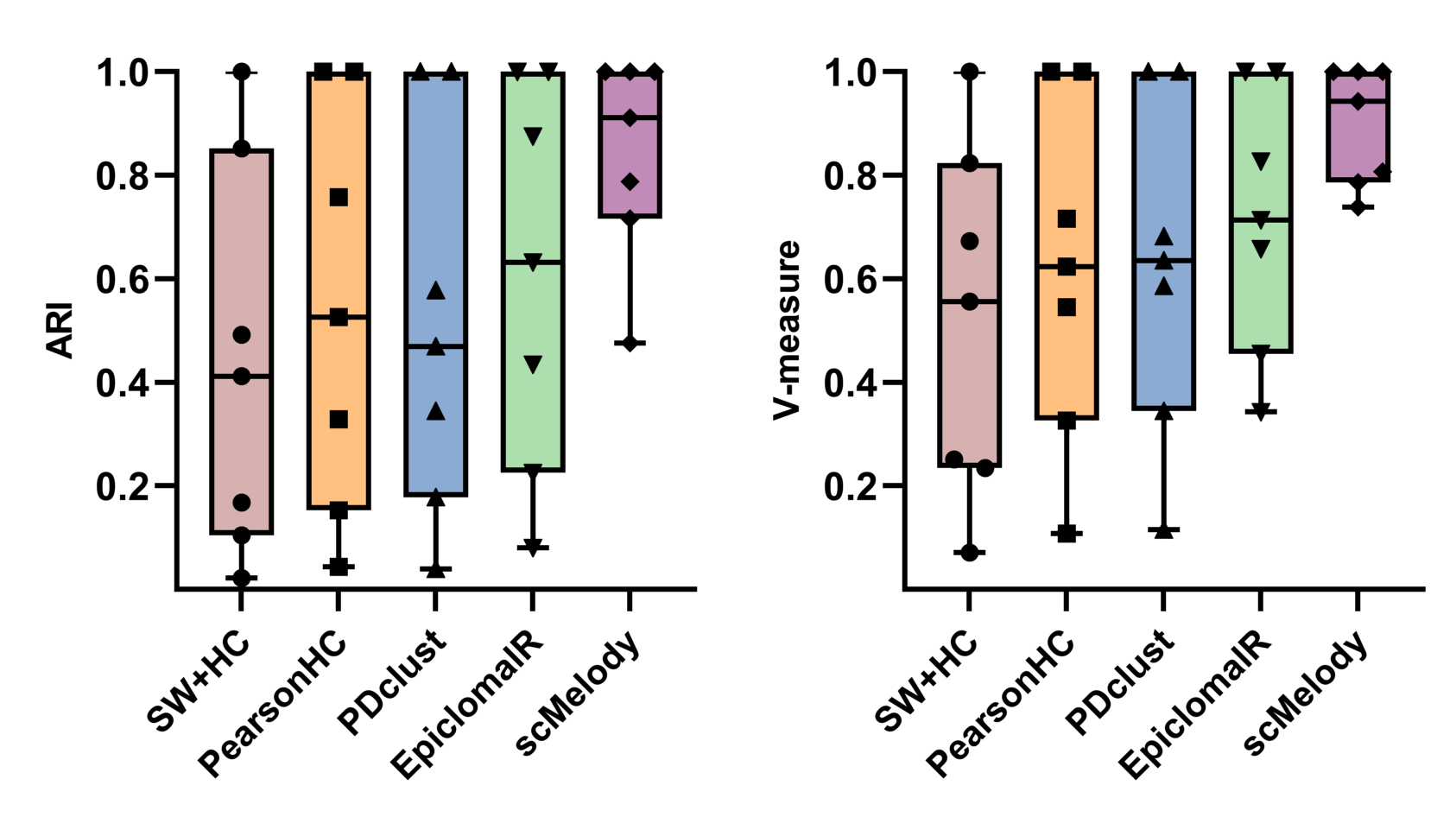


**Supplementary Figure 3.** Benchmarking scMelody and the major published methods across the real datasets. The consistency between the estimated and true cluster labels is measured through ARI and V-measure for the 7 different datasets. Each point in the boxplot represents the specific ARI or V-measure score of the corresponding method on the real dataset.


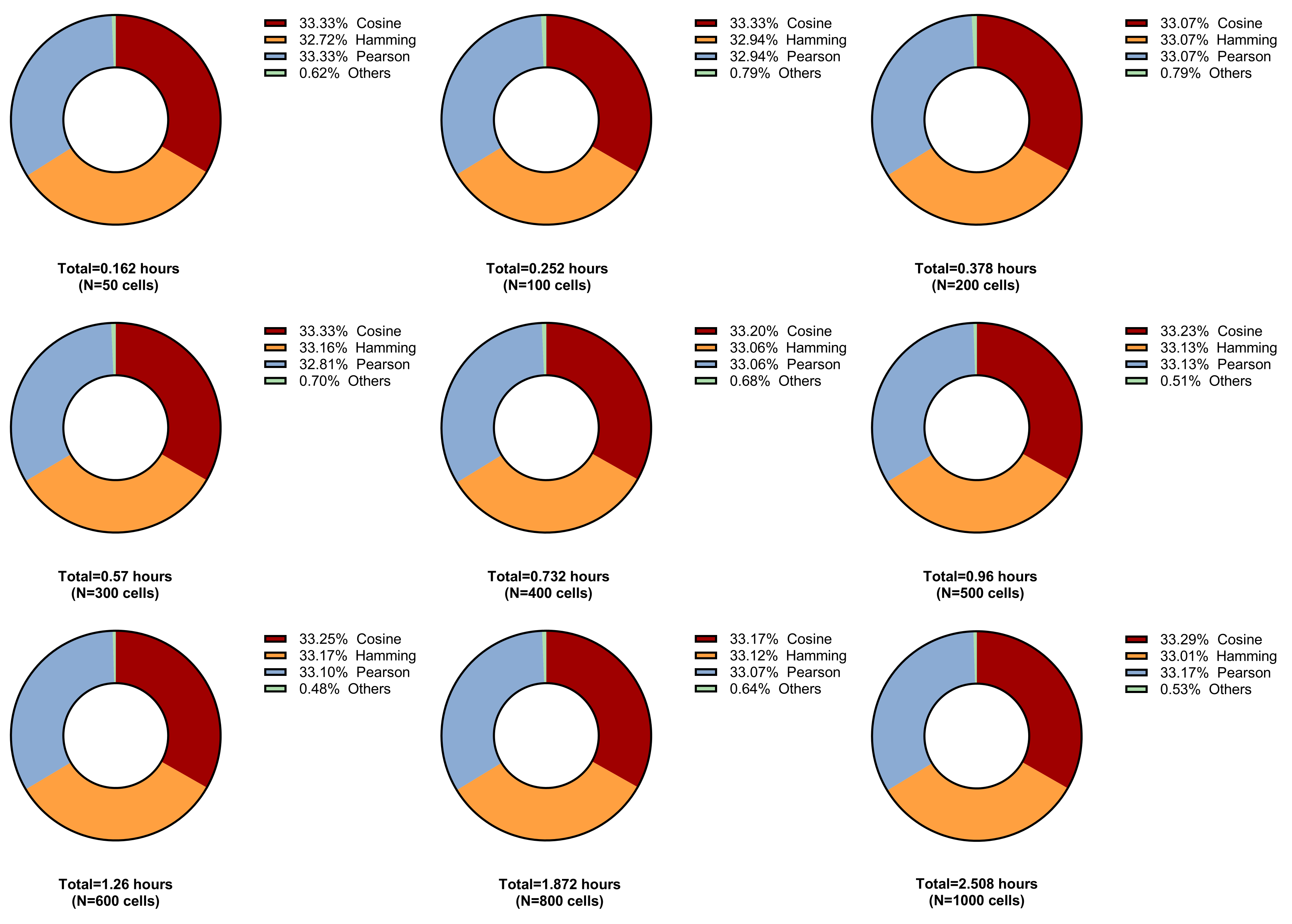


**Supplementary Figure 4.** The composition of the runtime of scMelody on the synthetic datasets with different cell numbers. For example, in the last subplot, we can find out that the total average runtime of scMelody is 2.508 hours while the calculation of the Cosine similarity matrix takes up 33.29% of the total runtime, the Hamming similarity matrix takes up to 33.01%, the Pearson similarity matrix takes up to 33.17% and other calculation steps of scMelody only take up to 0.53%.


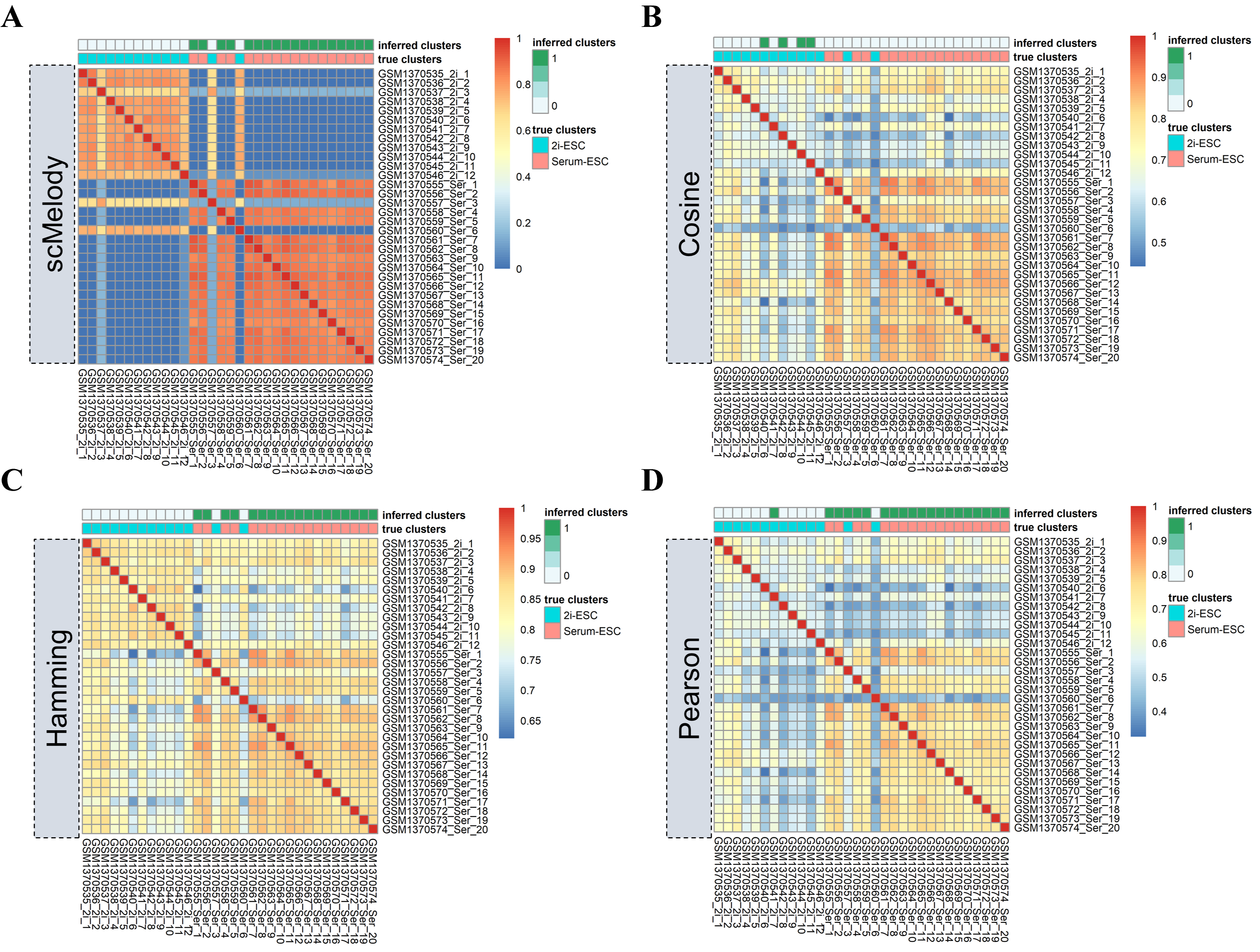


**Supplementary Figure 5.** Heatmaps of the different pairwise similarity measures for the Smallwood dataset, including the reconstructed similarity measure of scMelody (A), the Cosine similarity measure (B), the Hamming similarity measure (C) and the Pearson similarity measure (D).


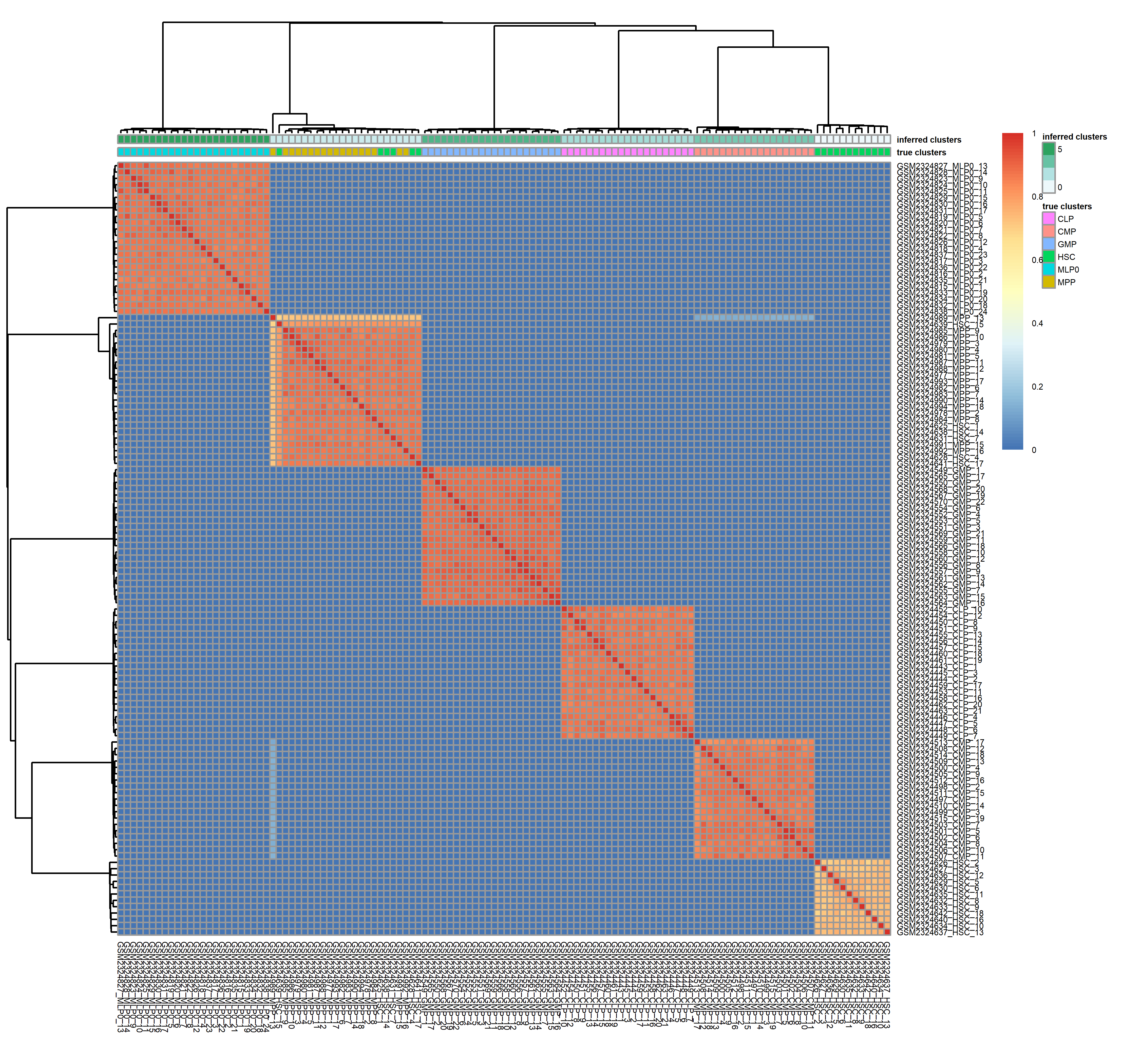


**Supplementary Figure 6.** Hierarchical clustering heatmap of the reconstructed similarity matrix for the Farlik2016 dataset. The inferred cell clusters are generated by the implementing the complete-linkage HC on the reconstructed similarity matrix.


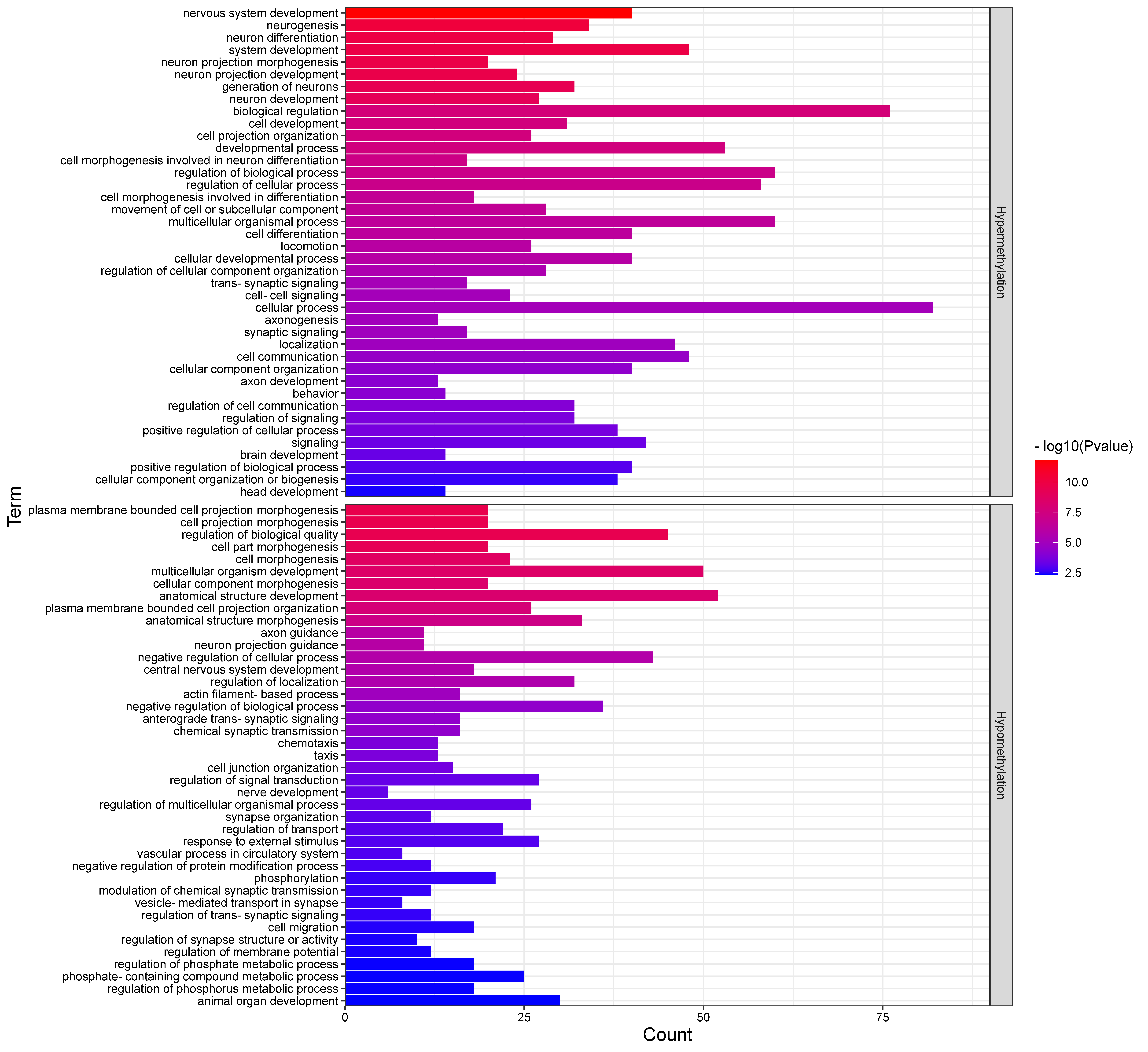


**Supplementary Figure 7**. Gene Ontology (GO) analysis of the differentially methylated genes (DMG) between the PAL-Inh novel and PAL-Inh Meis2. The DMGs are grouped by the average methylation levels between the two cell subpopulations, with the hypermethylation GO terms associated with higher methylation levels of DMGs in PAL-Inh novel subpopulation and hypomethylation GO terms associated with lower methylation levels of DMGs in PAL-Inh novel subpopulation.
